# Development of substituted benzylidene derivatives as novel dual cholinesterase inhibitors for Alzheimer's treatment†

**DOI:** 10.1039/d3ra03224h

**Published:** 2023-09-04

**Authors:** Shraddha Manish Gupta, Ashok Behera, Neetesh K. Jain, Avanish Tripathi, Dinesh Rishipathak, Siddharth Singh, Nafees Ahemad, Meryem Erol, Devendra Kumar

**Affiliations:** a Faculty of Pharmacy, Oriental University Indore 453555 Madhya Pradesh India; b Department of Pharmaceutical Sciences, School of Health Sciences and Technology, University of Petroleum and Energy Studies (UPES) Dehradun 48007 India; c Faculty of Pharmacy, School of Pharmacy and Population Health Informatics, DIT University Makkawala Dehradun Uttarakhand India; d Institute of Pharmaceutical Research, GLA University Mathura 281 406 U.P. India; e Department of Pharmaceutical Chemistry, MET's Institute of Pharmacy Nasik Maharashtra India; f School of Pharmacy, Monash University Jalan Lagoon Selatan, Bandar Sunway Petaling Jaya 47500 Selangor DE Malaysia; g Department of Pharmaceutical Chemistry, Faculty of Pharmacy, Erciyes University Kayseri Turkey; h School of Pharmacy & Technology Management, SVKM's NMIMS (Deemed-to-be) University Mukesh Patel Technology Park Shirpur 425405 India devendrak.phe@gmail.com +91 542 368428 +91 9455714362

## Abstract

Leading pathological markers of Alzheimer's disease (AD) include Acetylcholinesterase (AChE), Butyrylcholinesterase (BuChE), Amyloid beta (Aβ) and reactive oxygen species (ROS). Indole derivatives were identified and optimized to improve the potency against AChE, BuChE, Aβ and ROS. The lead molecule IND-30 was found to be selective for AChE (selectivity ratio: 22.92) in comparison to BuChE and showed maximum inhibition potential for human AChE (IC_50_: 4.16 ± 0.063 μM). IND-30 was found to be safe on the SH-SY5Y cell line until the dose of 30 mM. Further, molecule IND-30 was evaluated for its ability to inhibit AChE-induced Aβ aggregation at 0.5, 10 and 20 μM doses. Approximately, 50% of AChE-induced Aβ aggregation was inhibited by IND-30. Thus, IND-30 was found to be multitargeting for AD.

## Abstract 2

Leading pathological markers of Alzheimer's disease (AD) include Acetylcholinesterase (AChE), Butyrylcholinesterase (BuChE), Amyloid beta (Aβ) and reactive oxygen species (ROS).

## Graphical abstract

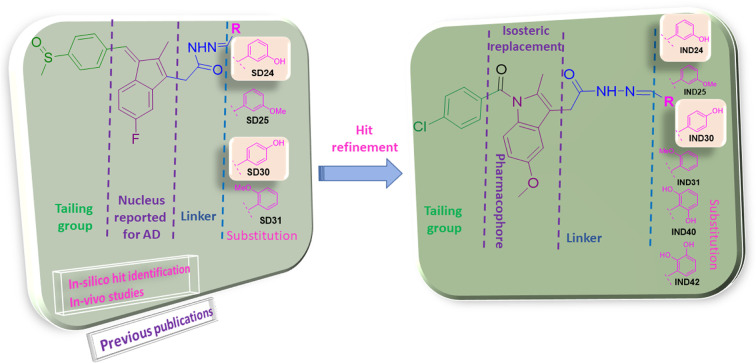

## 1 Introduction

Alzheimer's disease (AD) is a multifactorial and complex chronic neurological disorder that starts with memory loss, impairment of behavior and deficit in cognitive ability leading to death. The primary causes of the disease are the decline in neurotransmitter acetylcholine (ACh) levels, Amyloid-β (Aβ) plague formation, oxidative stress, dis-homeostasis of bio-metals and thin protein hyperphosphorylation followed by accumulation. According to the cholinergic hypothesis, AD is caused by the loss of the neurotransmitter ACh. Acetylcholinesterase (AChE) is the enzyme that degrades ACh. The availability of ACh is feasible by inhibiting the AChE enzyme. Several synthetic drugs are available in the market for the treatment of AD, but these drugs present side effects such as dizziness, nausea, vomiting, gut disturbance and hepatotoxicity. Drugs donepezil, galantamine and rivastigmine are used in the treatment of early stages of AD disease but have hepato-toxicity and gastrointestinal tract disorders as side effects. Thus, it is important to design new compounds with fewer side effects.

The active site of AChE/BuChE is divided into two subsites. The first is the catalytic anionic site (CAS) composed of a catalytic triad, an oxy-anionic hole and a cholinergic binding site composed of below mentioned amino acids. The second subsite is the peripheral anionic site (PAS) consisting of Tyr-72, Tyr-124, Asp-74, Tyr-341 and Trp-286 amino acid residues. However, Tyr-332 and Asp-70 are considered BuChE peripheral sites. The difference in the amino acid at the CAS and PAS indicates the selectivity of compounds such as phosphorous/acetates/alcohols/carbamides for either cholinesterase. The AChE PAS is involved in the formation of the stable AChE-Aβ complex that is more toxic than age-related Aβ peptide aggregates. For this reason, inhibitors able to interact with CAS and PAS at the same time and the same amino acids are known as dual site binding inhibitors classified as multi-target drugs. Furthermore, oxidative stress and aging contribute a large part of the pathophysiological mechanism associated with AD.

**Fig. 1:**
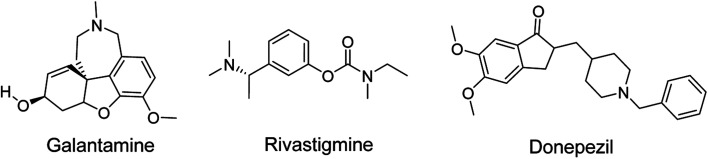
FDA approved acetyl cholinesterase inhibitors (AchEIs) in AD.

Heterocyclic synthesis has evolved into a potent approach for the production of novel compounds for drug discovery and development. Heterocyclic compounds containing nitrogen provide highly functionalized scaffolding for the development of potent and selective pharmaceuticals.^1–3^ ACh is clinically relevant in numerous disease processes, the most prevalent of which are AD, Lambert–Eaton myasthenic syndrome (LEMS), and myasthenia gravis (MG). The major neurotransmitter system implicated in the etiology of AD is the cholinergic group of neurons, which is involved in cortical activity, cerebral blood circulation, memory and learning-oriented processes, and modulation of cognition.^4^ It is a progressive neurodegenerative illness that affects the central nervous system of aged persons.^5^ AD patient brains exhibited specific depletion of the choline acetyl transferase enzyme, which reduces acetylcholine production and impairs cortical cholinergic function. Cholinesterase inhibitors, such as donepezil, rivastigmine, and galantamine (Fig. 1), enhance cholinergic transmission by inhibiting cholinesterase at the synaptic cleft.^6,7^ To date, there are no viable treatments for Alzheimer's disease.^8^

**Fig. 2:**
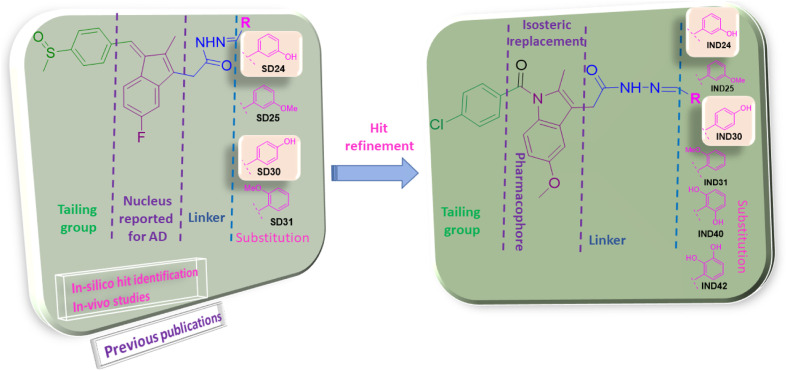
Rational of designing of indole analogs followed by synthesis, characterization, and biological screening.

Previous reports suggested that indene methylene analogs were active for the AD.^9^ Thus, the library of the indene analogs was prepared and detailed *in silico* analysis was reported in our previous publication.^10^ Further, our next publication reported the *in vitro* analysis of the indene methylene analogues.^11^ The encouraging results in these studies showed indene nucleus attached to the linker, substituent and tailing group are vital for the activity. Hydroxy substitution at *para* and *ortho* positions was found to be most active against AChE.^11^ Thus, hit refinement was performed to improve the AChE, BuChE, ROS scavenging and Aβ inhibition property of the compounds. The isosteric replacement drug design approach suggested that hit molecules should be re-engineered to enhance efficacy.^12^ So, indene was replaced with its isostere indole nucleus. Notably, the docking interaction between the indole nucleus and the AChE was found to be improved. Further, replacing the hydrazine linker with the carbon chain resulted in the loss of interactions. However, replacing methylsulfinyl-4-benzene with 4-chlorophenylethanone from the tailing group was found to be suitable for the activity. Finally, the substitution attached to the linker was optimized. Various functional groups attached to the *ortho*/*meta*/*para* positions of benzene as substituents are evaluated. Electron withdrawing groups *viz.*, nitro, carboxy *etc.*, and halogens were substituted at the various positions of the benzene ring. Molecular electrostatic potential (MEP) analysis depicted that electron withdrawing substitutions and halogens are unfavorable for chemical reactions and intermolecular interactions. Further, docking analysis also revealed that these groups decrease the interaction with the active site of AChE and BuChE. However, hydroxy and methoxy groups were found to be favorable for the interaction in docking analysis. MEP analysis also represented its synthetic feasibility and intramolecular interaction. Along with the enzyme inhibition and synthetic feasibility, the BBB permeability of the compounds was the limiting factor. *In silico* BBB permeability study was performed, and molecules having methoxy and hydroxy substitutions are found to be brain permeable. Thus, previous studies and *in silico* analysis of indole analogues become the basis for the selection of molecules for synthesis (Fig. 2).

The process of identifying the potent indole analogs includes the rigorous *in silico* approach based on our previous publications. The library of 44 Indomethacin analogs was prepared. Various electron donating and withdrawing groups were used as substituents. The created library was subjected to docking analysis on AChE. Further, these analogs were subjected to *in silico* ADME, drug-likeness and toxicity analysis. Based on the binding pose analysis, docking score and ADME properties six molecules were selected for supplementary *in silico* analysis. BBB permeability is the prerequisite property of the molecules to be active for AD. The molecular weight of the drug candidates should be below 500 Da, *c* log *P* should be in the range of 2–5 and hydrogen bond donors (HBD) are accepted less than three. Further, selected molecules have a molecular weight below 500, hydrogen bond donor 2–3. These structural properties are vital for the drug to act on AD.^13^ The binding of designed molecules with the CAS and PAS site of AChE are important for activity. The docking analysis showed the binding of the selected Indole analogs with these sites of AChE. The presented research work reported the design, and synthesis of potential indole derivative screened by virtual tools, followed by *in vitro* AChE and BuChE inhibition assays, antioxidant activity, cell line-based toxicity study on SH-SY5Y cell line and AChE-induced Aβ_1–42_ aggregation assay (ESI, Fig. S1†).

## 2 Results and discussion

### 2.1 Design, synthesis, and spectroscopic properties

**Scheme 1:**
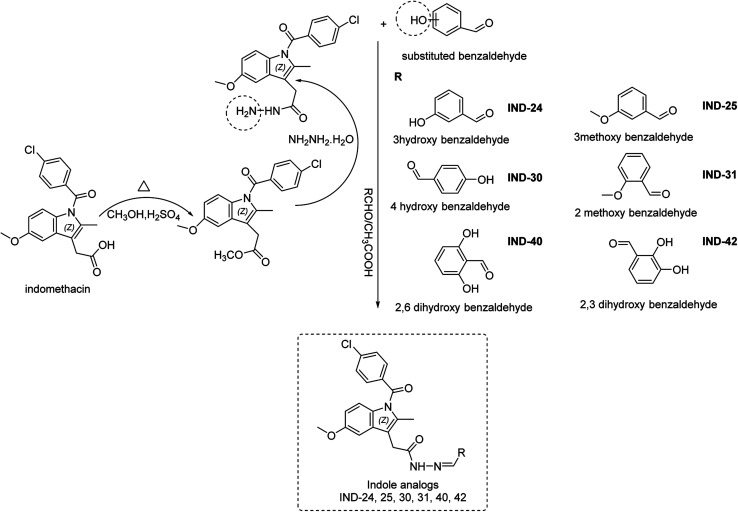
Synthesis of indole derivatives.

Indomethacin is a methylated indole derivative consisting of indole moiety. ChemDraw 19.1 was utilized to construct a unique library of 44 methylated indole analogs by swapping R with electron donor and acceptor groups. Based on computational screening, six lead compounds were derived,^14^ and synthesized (as shown in Scheme 1). The typical steps were esterification, hydrazide derivatives, and lastly coupling reaction between different aldehydes.^15^ Each product was purified by column chromatography and characterized by FTIR, ^1^H NMR, ^13^C NMR, and HRMS spectra.

### 2.2 Computational studies

#### 2.2.1 Molecular docking, binding energy calculations

**Fig. 3:**
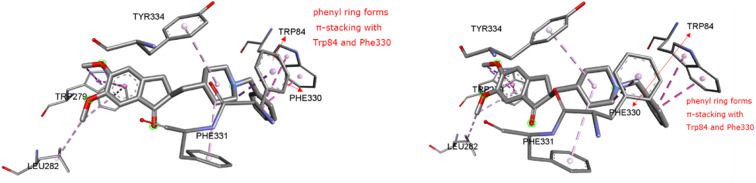
Ligand binding sites of target protein structure (1EVE) chain A with standard E2020.

**Fig. 4:**
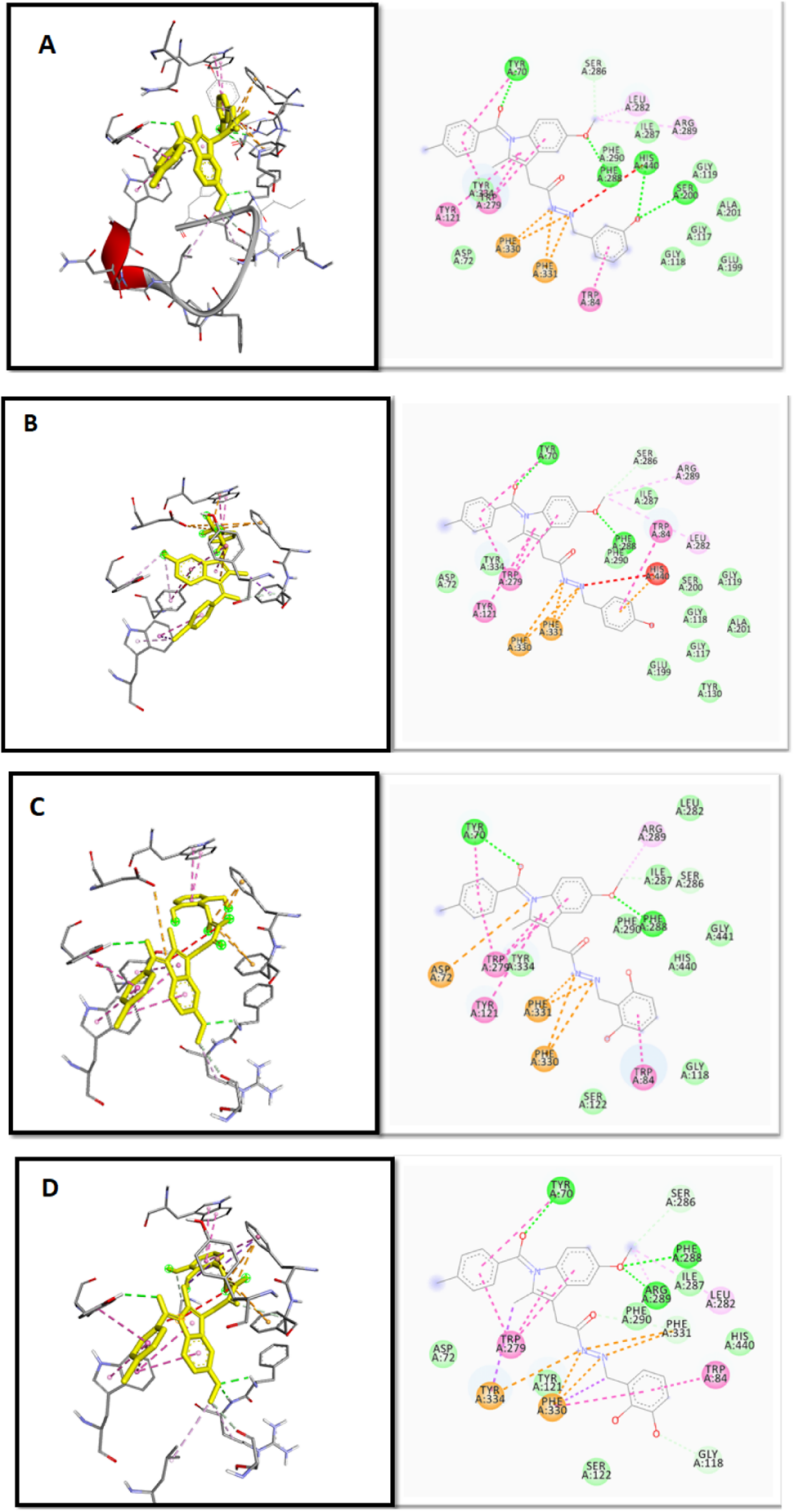
2D and 3D binding mode and chemical interactions of the best four lead molecules with the target protein. (A): IND-24 (docking score: −11.4); (B): IND-30 (docking score: −11.4); (C): IND-40 (docking score: −11.1); (D): IND-42 (docking score: −11.3).

To validate the docking protocol, an X-ray ligand, donepezil (E2020) was first docked into the binding pocket. It was found that the donepezil sits exactly in the same position, and the RMSD was found to be 1 Å, confirming the precision and reproducibility of the docking method. As revealed by G. Kryger *et al.*,^16–19^ E2020's phenyl ring forms stacking with Trp84 and Phe330, while another aromatic ring forms stacking with Trp279. Additionally, a hydrogen bond was discovered between Phe288 and ketone oxygen (Fig. 3). All analogs exhibit favorable docking energy scores and chemical interactions relative to the standard donepezil (ESI Table S1† and Fig. 4). AutoDock Vina demonstrated an abundance of hydrogen and hydrophobic interactions between ligands and targets (Table 1).

**Table 1:** Docking scores by Auto Dock Vina and interactive residues of best 3 ligands with chain A of 1EVE

ID	Docking score (kcal mol^−1^)	Interactive residues chain A
IND-24	−11.4	TRP A: 84, PHE A: 288, TYR A: 334, PHE A: 330, ARG A: 289, ASP A: 72
IND-25	−11.3	TRP A: 84, PHE A: 330, PHE A: 331, TYR A: 334, TRP A: 279, TYR A: 70, ARG A: 289
IND-30	−11.4	TRP A: 84, PHE A: 331, TYR A: 334, TRP A: 279, TYR A: 70, PHE A: 330, ARG A: 289
IND-31	−11.4	TRP A: 84, PHE A:331, TYR A: 334, TRP A: 279, TYR A: 70
IND-40	−11.1	TRP A: 84, PHE A: 288, PHE A: 330, ARG A: 289, TYR A: 121, HIS A: 440, SER A: 122, ASP A: 72
IND-42	−11.3	TYR A: 70, PHE A: 331, PHE A: 330, TYR A: 334, LEU A: 282, GLY A: 118, GLY A: 119, SER A: 200, HIS A:440
E2020	−11	TRP A: 84, PHE A: 330, PHE A: 331, TYR A: 334, TRP A: 279, TYR A: 70, PHE A: 288, SER A: 286

#### 2.2.2 Molecular dynamics simulation

**Fig. 5:**
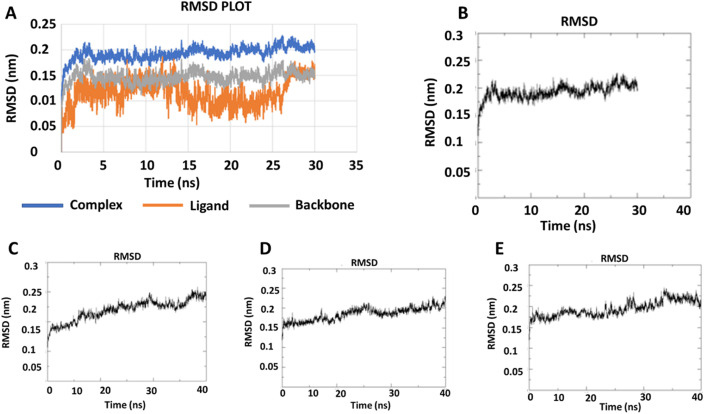
RMSD trajectory for (A) the macromolecular target as well as a ligand (IND-24) and (B) IND-24, (C) IND-30, (D) IND-40, (E) IND-42 obtained after performing molecular dynamics simulation.

**Fig. 6:**
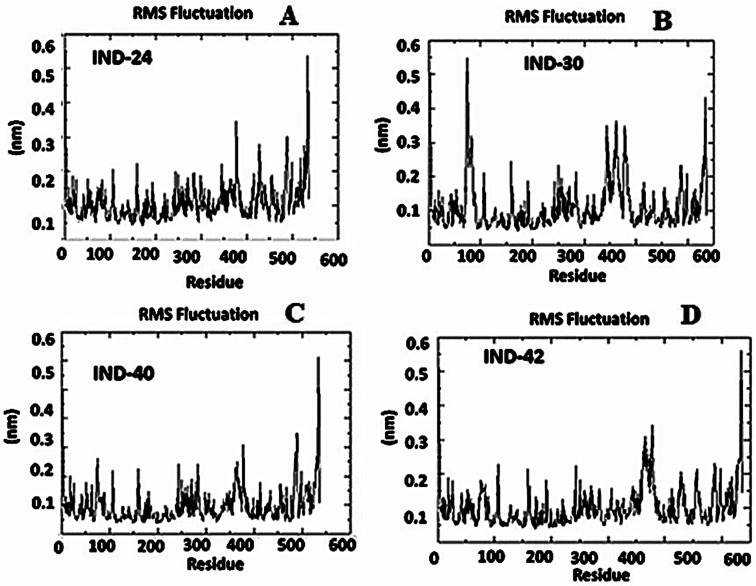
Root mean square fluctuation (RMSF) analysis of the apo form, (A) IND-24, (B) IND-30, (C) IND-40 and (D) IND-42 compound holo form of acetylcholinesterase (AchE) throughout 30 ns (PDB ID: 1EVE).

**Fig. 7:**
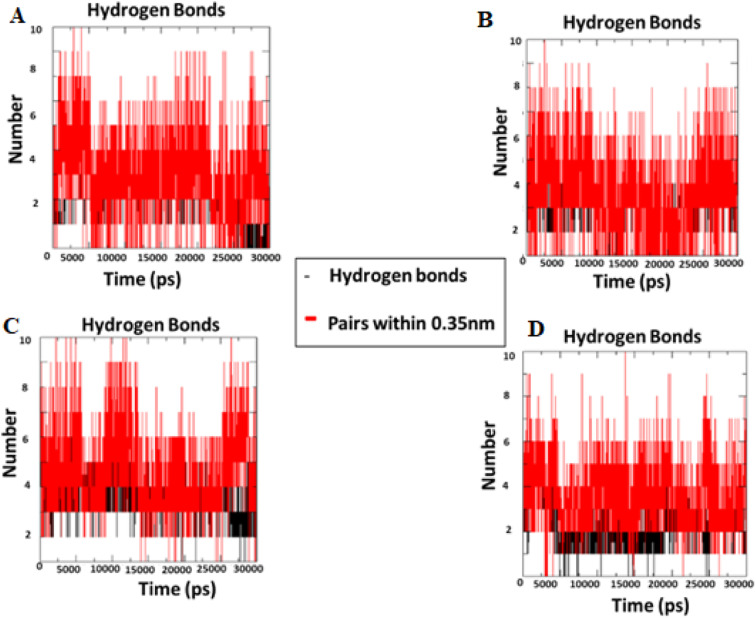
Intermolecular H bonds number between acetylcholinesterase – (A) IND-24, (B) IND-30, (C) IND-40 and (D) IND-42 compounds for 30 ns.

During the simulation time, the RMSD technique assesses the stability and structural changes in the protein backbone. We found that complicated docked trajectories were stable throughout the simulation, with a mean RMSD value of 4 (Fig. 5). It indicates a steady behavior with low variance. The complex was initially stable up to 16 ns, after which there was a minor deviation between 17 and 26 ns, after which it almost remained there projecting a near about static equilibrium, and as a result, the trajectory was found to be very stable (RMSD 0.15 nM, 1.5 Å). To gain insight into the structural fluctuations of active amino acid residues in the AChE enzyme, we estimated the RMSF values using the C atoms of IND-24, IND-30, IND-40, and IND-42 molecules from the stable trajectory. The mobility of amino acids in the active site decreases as variations decrease, and *vice versa*. The amino acid residues are plotted on the *x*-axis and their RMSF value is on the *y*-axis in RMSF (Fig. 6). The complex system's active amino acid residues were analyzed and found to have a lower RMSF value. The average fluctuation range was found to be 1.2–1.8 Å for the IND-24, IND-30, IND-40, and IND-42. As a result, RMSF analysis of a few complexes revealed similar changes in active site residues that are crucial for molecular interactions. During the MD simulation, the formation of hydrogen bonds, hydrophobic interactions, and ionic interactions between the protein–ligand complex is responsible for their stability. As a result, we quantified the stability of our screened compounds against the enzyme AChE by evaluating the strength of these interactions. The information-rich feature is always represented by the number of hydrogen bonds. The plot (Fig. 7) between time in picoseconds on the *X*-axis and the number of hydrogen bond contacts on the *Y*-axis between protein and ligand revealed that nearly two hydrogen bonds were observed in the docked complexes of IND-24 and IND-30, whereas an average of three hydrogen bonds were observed in IND-40 and IND-42.

#### 2.2.3 MEP analysis and geometric optimization

**Fig. 8:**
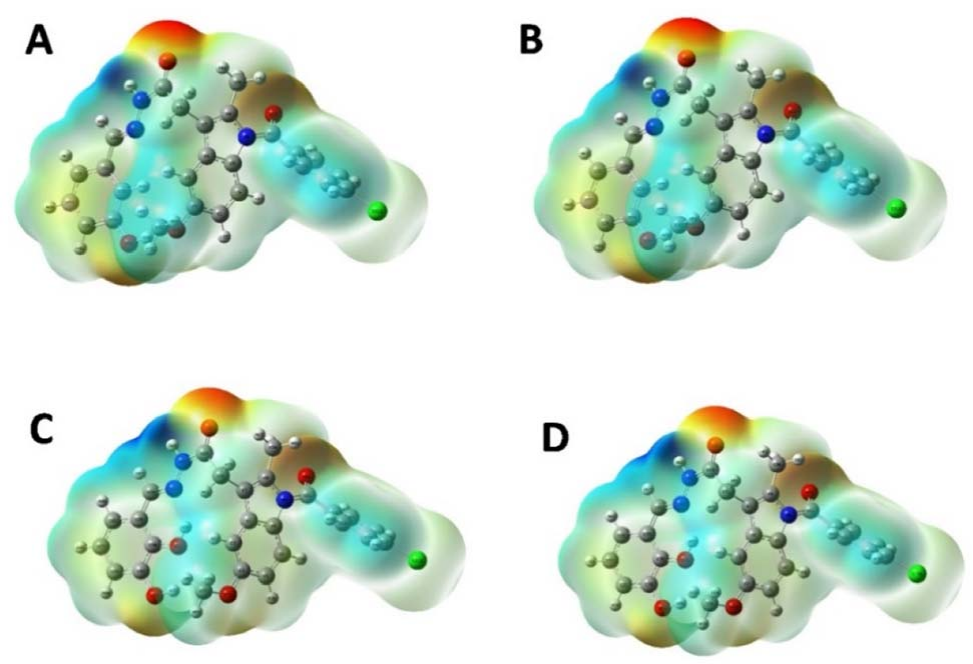
MEP plots of (A) IND-24; (B) IND-30; (C) IND-40 and (D) IND-42.

The MEP is based on the interaction energy between the molecular charge distribution of the system and the positive unit charge and is also a combination of contributions from both the electronic distribution and the nucleus.^20^ It also plays a very important role in the determination of chemical reactions and intermolecular interactions.^21^ In the MEP map, the region with the lowest electron density is blue, the region with the highest electron density is red, and the potential increases are listed as red < orange < yellow < green < blue.^22^ The MEP surface map of IND-24, IND-30, IND-40, and IND-42 are calculated according to the DFT/B3LYP/6-311G(d) basis set was shown in Fig. 8. Negative regions of MEP (red) are associated with electrophilic reactions, while positive regions of MEP (blue and green) are associated with nucleophilic reaction sites.^23^ The blue and green regions are mainly concentrated on carbon and hydrogen atoms, and the red and yellow regions are concentrated on oxygen and nitrogen atoms.

#### 2.2.4 Molecular reactivity analysis

**Fig. 9:**
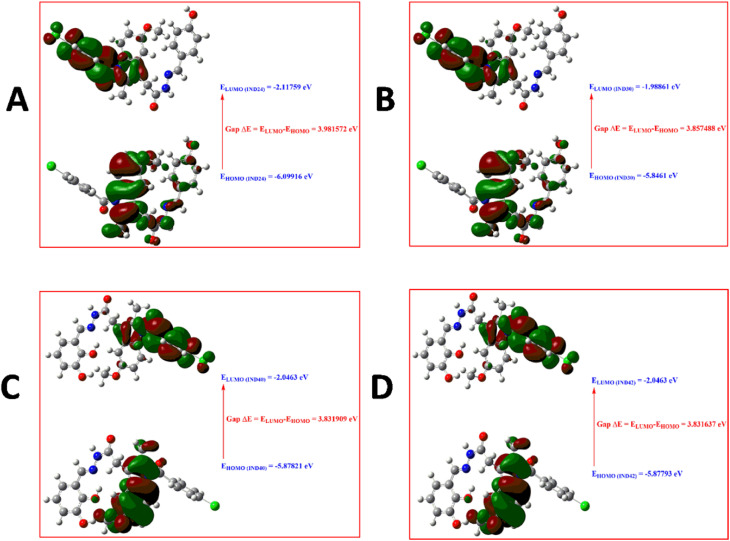
HOMO–LUMO diagram of (A) IND-24; (B) IND-30; (C) IND-40 and (D) IND-42.

Density functional theory is an important component of research in computing quantum mechanical ground states of molecular systems. The development of sufficiently accurate functionals, efficient algorithms, and continuous improvements in computing capabilities have further increased the importance of DFT.^24^ Since its inception, DFT has become more useful than ever for calculating the ground state energy of molecules/solids of any system containing electrons and nuclei.^25^ The use of DFT on bioactive compounds has assisted in the design of new compounds with therapeutic effects, where they gained a deeper understanding of their effects.^26^ DFT calculations provide important insights into the physicochemical properties, reaction sites and possible effects of the studied compounds. The appropriate methodology for scientific research, which provides important tools for the analysis and prediction of physicochemical parameters and/or biological activity, is the molecular modeling method and can be defined as the sum of the theoretical methods and computational techniques used to investigate the behavior of the molecule.^27^ Frontier molecular orbitals, known as the highest occupied molecular orbital (HOMO) and the lowest unoccupied molecular orbital (LUMO), are very popular quantum chemical descriptors. They play an important role in the formation of many charge transfer complexes and in controlling many chemical reactions. The majority of the chemical reaction takes place when the HOMO and LUMO overlap of the reactants is the maximum.^28^ Boundary orbitals of molecules, HOMO and LUMO, are mostly used to talk about chemical reactions in molecules. Since most chemical reactions take place by electron exchange, HOMO–LUMO and some other parameters derived from them directly affect the chemical behavior of the molecule. HOMO is defined as donating an electron, while LUMO is defined as accepting electrons.^20^ This concept is used only when describing the reactivity of different atoms in the same molecule, but for comparing the reactivity of atoms in different molecules, the energy level of HOMO reflects the relative reactivity of different molecules. The HOMO energy is directly related to the ionization potential and indicates the molecule's susceptibility to attack by electrophiles. LUMO energy is related to electron affinity and indicates the susceptibility of the molecule to attack by nucleophiles.^28^ Briefly, these transitions can be described as HOMO to LUMO. IND-24 has a high electron-accepting tendency, and IND-30 has a higher electron donation. Also, the larger the energy difference (Gap Δ*E*: LUMO–HOMO), the more stable the molecule. The most stable compounds were listed as IND-24 > IND-30 > IND-40 > IND-42 respectively. The HOMO–LUMO energy differences and all other parameters calculated from these energy differences were presented in Table 2. The localized states of IND-24, IND-30, IND-40, and IND-42 HOMO–LUMO energies are shown in Fig. 9, and it was seen that the substituted groups did not affect the HOMO and LUMO energy distribution.

**Table 2:** Calculated frontier molecular orbital parameters of IND-24, IND-30, IND-40 and IND-42^a^

Comp.	HOMO (eV)	LUMO (eV)	Gap Δ*E*	IP (−HOMO)	EA (−LUMO)	*η’* (IP − EA/)2	*M* − (IP + EA)/2	*S* 1/2*η’*	*X* (IP + EA)/2	*ω* (μ2/2*η*)
IND-24	−6.09916	−2.11759	3.981572	6.099163	2.117591	1.990786	−4.10838	0.251157	3.917353	2.11961
IND-30	−5.8461	−1.98861	3.857488	5.846097	1.988609	1.928744	−3.91735	0.259236	3.962252	1.989074
IND-40	−5.87821	−2.0463	3.831909	5.878207	2.046297	1.915955	−3.96225	0.260967	3.962116	2.048514
IND-42	−5.87793	−2.0463	3.831637	5.877935	2.046297	1.915819	−3.96212	0.260985	3.962052	2.048519

a Gap Δ*E*: LUMO–HOMO, IP (−HOMO): ionization potential, EA (−LUMO): electron affinity, *X* (IP + EA)/2: electronegativity, *η* (IP − EA/)2: chemical hardness, *S* (1/2*η’*): chemical softness, *μ* − (IP + EA)/2: chemical potential, *ω* (μ2/2*η*): electrophilic index.

### 2.3 AChE and BuChE inhibition assay

Due to the multifaceted nature of AD, the multifunctional molecules were developed to target the illness's underlying causes. Initially, the effect of hydroxy and methoxy groups on *in vitro* enzyme inhibition was evaluated. These groups were substituted to the *ortho*/*meta* and *para* positions of the benzylidene moiety. A hydrogen bond donor (HBD) was introduced at the *meta*/*para* position of the indole series of compounds to investigate the role of hydrogen bonding in the interaction with targets. Best hAChE inhibition (IC_50_ = 4.16 ± 0.063) potential was observed for the *para* hydroxy group-containing compound IND-30. The molecule IND-30 was found to be more selective for hAChE (selectivity ratio 22.92). When the hydroxy group was shifted to the *meta* position the inhibition potential was found to be decreased (IND-24, hAChE IC_50_ 12.54 ± 0.143, hBuChE IC_50_ 98.18 ± 0.120). IND-40 and IND-42 showed a significantly decreased in the inhibition potential of both enzymes (hAChE IC_50_ 70.54 ± 0.133, hBuChE IC_50_ 689.12 ± 0.128 and hAChE IC_50_ 122.75 ± 0.086, hBuChE IC_50_ 668.22 ± 0.133 respectively) (Table 3). The shift in the activity of compounds IND-40 and IND-42 may be due to increased hindrance inside the catalytic domain due to the presence of di-hydroxy groups. Further, the effect of methoxy substitution on various positions was assessed. AChE and BuChE inhibitions were further found to be declined (IND-25 hAChE IC_50_ 264.23 ± 0.102, hBuChE IC_50_ > 1000 and IND-31 hAChE IC_50_ 421.22 ± 0.086, hBuChE IC_50_ > 1000).

**Table 3:** Methylated indole derivatives with inhibitory activities (IC_50_) against AChE and BuChE and antioxidant activity^a^

Compound	*R*	IC_50_ ± SE (μM)			% free radicle scavenging, mean ± SE (μM)	
		hAChE	hBuChE	Selectivity ratio	50	100
IND-24	–3OH	12.54 ± 0.143	98.18 ± 0.120	7.83	17.68 ± 0.478	34.34 ± 1.333
IND-25	–3OCH_3_	264.23 ± 0.102	>1000	3.78	11.51 ± 0.748	27.99 ± 1.127
IND-30	–4OH	4.16 ± 0.063	95.34 ± 0.144	22.92	15.19 ± 0.881	30.65 ± 1.010
IND-31	–2OCH_3_	421.22 ± 0.086	>1000	2.37	9.75 ± 0.625	26.43 ± 0.887
IND-40	–2,6OH	70.54 ± 0.133	689.12 ± 0.128	9.77	24.10 ± 0.889	50.39 ± 0.930
IND-42	–2,3OH	122.75 ± 0.086	668.22 ± 0.133	5.44	18.21 ± 0.598	38.87 ± 1.012
DNP	—	0.03 ± 0.022	01.41 ± 0.084	—	—	—
Ascorbic acid	—	—	—	—	42.66 ± 1.491	76.76 ± 0.708

a Selectivity ratio = (IC_50_ of BuChE)/(IC_50_ ofAChE).

### 2.4 Evaluation of cell line-based toxicity study on SH-SY5Y cell line

**Fig. 10:**
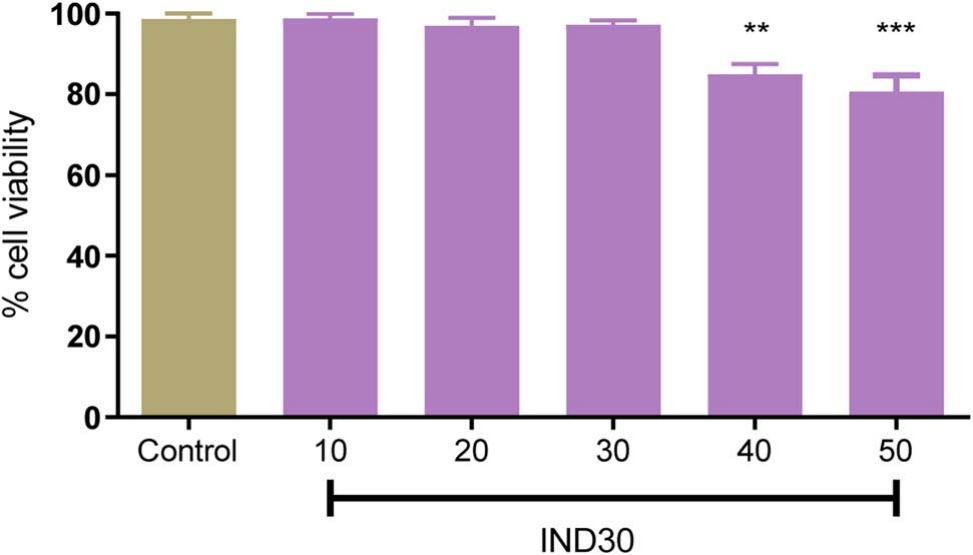
Cell toxicity study on SH-SY5Y cell line at five different doses. One Way ANOVA was used with multiple comparisons.

In recent decades, *in vitro* cell line research has become commonplace since they are useful for primary optimization and mechanistic investigations before toxicological studies on animals.^29^ The evaluation of toxicity of compound IND-30 (10, 20, 40, and 50 mM) on human neuroblastoma SH-SY5Y cell lines was assessed through MTT (3-(4,5-dimethylthiazol-2-yl)-2,5-diphenyl tetrazolium bromide) assay (Fig. 10). Compound IND-30 demonstrated no significant toxicity up to 30 mM. Thus, it was evident that the incubation of SH-SY5Y cell lines with compound IND-30 was safe.

### 2.5 AChE-induced Aβ_1–42_ aggregation assay

**Fig. 11:**
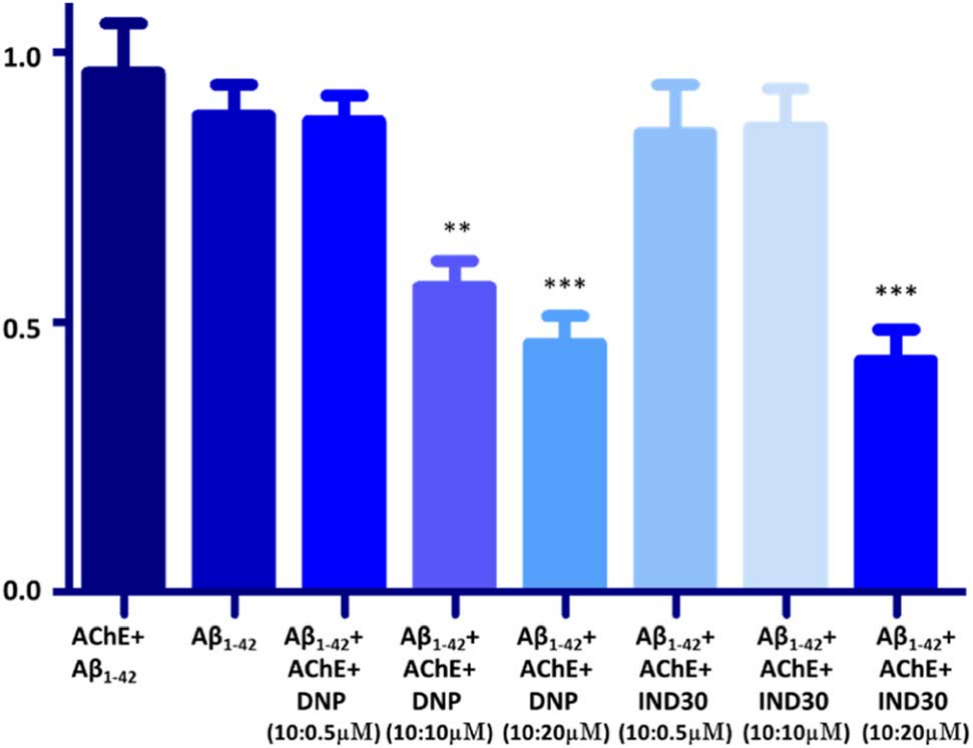
Effect of donepezil (DNP) and IND-30 on the AChE-induced aggregation of Aβ: DNP, IND-30 decreased Aβ aggregation in a concentration-dependent manner; AChE-induced Aβ aggregation is decreased by DNP and compound IND-30 (One-way ANOVA followed by one-way analysis of variance***p 0.0001, compared with AChE + Aβ_1–42_), error bars depict the standard deviation (SD) of the normalized fluorescence intensity (NFI).

AChE enzyme is reported to facilitate the aggregation of Aβ. Thus, the potent compound IND-30 was evaluated for its ability to inhibit AChE-induced Aβ aggregation. Aβ_1–42_ and AChE treated Aβ_1–42_ are 100% aggregated after the incubation of 48 h. The marketed compound donepezil showed a significant decrease in the Aβ_1–42_ aggregation at 10 and 20 μM. Further, the compound IND-30 was found to be effective only at the higher dose (20 μM) (Fig. 11).

### 2.6 Structural activity relationship

Previous in-house publications revealed the significance of Indene moiety in interaction with AChE and BuChE^30^ (Fig. 2). Isosteric replacement of the indene with indole was performed to improve the interaction. Various tailing groups, linkers and substituents were explored to improve the interactions. Hydroxy and methoxy containing benzaldehyde as substituents were selected for the synthesis based on *in silico* studies. *In silico* studies revealed that replacing Chloro benzene at the tailing group with other halo benzenes decreases the activity. Further, an indole ring with acetohydrazide as a linker was found to be essential for activity. Thus, substituted benzaldehyde was used to regulate the interaction of molecules with AChE and BuChE. IND-24 having a *meta* hydroxy group had a docking score of −11.4 kcal mol^−1^. The molecule retained the interaction with TRP A: 84 and TYR-A: 334 amino acid in the A chain of AChE as showed by natural inhibitor E2020 (Table 1). The hAChE and hBuChE inhibition potential of the compound was found to be 12.54 ± 0.143 and 98.18 ± 0.120 μM respectively. The compound was found to be selective on AChE (selectivity ratio 7.83). Further, the *meta* methoxy group (IND-25) was introduced based on docking results. IND-25 retained most of the amino acid residue interactions with the AChE (Table 1). However, its AChE inhibition potential decreases significantly (264.23 ± 0.102 μM) and the compound was found to be inactive on the BuChE enzyme. Moving forward the hydroxy group is shifted to the *para* position (IND-30). The molecule retained most of the interactions as shown by E2020. IND-30 has the highest hAChE and BuChE inhibition potential (4.16 ± 0.063 and 95.34 ± 0.144 μM respectively) and was found to be selective for hAChE (22.92). IND-31 having methoxy group at *ortho* position has the lowest hAChE inhibition potential and is inactive on hBuChE (421.22 ± 0.086 μM). Thus, the substitution of methoxy group on *ortho*/*meta*/*para* positions was found to decrease AChE and BuChE inhibition potential. As methoxy substitution was found to be active. Thus, dimethoxy-containing compounds were synthesized (IND-40, IND-42). Steric hindrance and unfavorable interactions in the catalytic site of AChE and BuChE decrease the inhibition potential of the compounds. Thus, the hydroxy group at *meta* and *para* positions are favorable for activity. However, the dihydroxy group was found to decrease the activity. The Methoxy group at any position was found to be unfavorable for the activity.

## 3 Conclusion

The indole derivatives library was prepared based on previous reports. Further, the library was screened using *in silico* techniques for its AChE inhibition potential, drug-likeness and ADME properties. Finally, the selected molecules (IND-24, IND-25, IND-30, IND-31, IND-40 and IND-42) were synthesized, characterized and *in vitro* experiments were performed. The AChE binding potential of the selected compounds IND-24 and IND-30 was found to be 11.4 kcal mol^−1^. The stability of the compounds was predicted before synthesis by MEP and molecular reactivity analysis. The stability of the compounds is listed as IND-24 > IND-30 > IND-40 > IND-42 respectively.

Human AChE and BuChE inhibition potential of the compounds were assessed with reference to donepezil. IND-30 was found to be the most potent compound having human AChE and BuChE inhibition potentials of 4.16 ± 0.063 and 95.34 ± 0.144 μM. However, the IND-30 was found to be 22.92-fold more selective for BuChE. IND-24 is the next potent compound with AChE and BuChE inhibition potential of 12.54 ± 0.143 and 98.18 ± 0.120 μM. These compounds were found to be active due to the presence of the hydroxy group. Thus, two hydroxy groups were substituted on the benzylidene to assess the enzyme potential (IND-40, IND-42). Unsuccessfully, the IC_50_ of the compounds further dwindled (IND-40 = 70.54 ± 0.133 μM AChE, 689.12 ± 0.128 μM BuChE, IND-42 = 122.75 ± 0.086 μM AChE, 668.22 ± 0.133 μM BuChE). Further, the methoxy group was substituted on the benzylidene (IND-25 and IND-31). However, the substitution was found to be unfavorable and inhibition potential further decreases (IND-25 = 264.23 ± 0.102 μM AChE, >1000 μM BuChE, IND-31 = 421.22 ± 0.086 μM AChE, >1000 μM BuChE). The AChE and BuChE inhibition pattern showed that hydroxy substitution at the benzylidene ring is favorable for activity. However, activity is maximum when a single hydroxy group is substituted at the fourth position.

Further, the antioxidant potential of the compounds was assessed by DPPH assay at 50 and 100 μM. As expected, compounds IND-40 and IND-42 showed maximum antioxidant potential (24.10 ± 0.889% and 18.21 ± 0.598%). The higher antioxidant potential of these compounds is due to the presence of two hydroxy groups. Assessment of the toxicity is the prerequisite study for the synthetic derivatives. The toxicity of the most potent compound (IND-30) was evaluated on the neuroblastoma SH-SY5Y cell lines. AChE enzyme provides the site for the aggregation of the Aβ_1–42_. Thus, AChE-induced Aβ_1–42_ aggregation was assessed for compound IND-30. IND-30 was found to be active at 20 μM on AChE induced Aβ_1–42_ aggregation assay.

## 4 Experimental section

### 4.1 Material and methods

Indomethacin sample obtained as a gift from the hetero lab, Hyderabad. Further, methanol, sulfuric acid, hydrazine hydrate, ethanol, acetic acid, 3-hydroxy benzaldehyde, 4-hydroxy benzaldehyde, 2-methoxy benzaldehyde, 3-methoxy benzaldehyde, 2,3-dihydroxy benzaldehyde, 2,6-dihydroxy benzaldehyde were obtained from commercial sources (Himedia, Sigma-Aldrich) of the highest analytical grade and used without further purification unless otherwise stated. Uncorrected checks of melting points utilizing open capillary procedures on a Macro scientific instrument was performed. All reactions were routinely analyzed using thin-layer chromatography (TLC) on silica gel G plates eluted with a mixture of ethyl acetate and petroleum ether (5 : 5) as the solvent system. Using a Shimadzu FTIR spectrophotometer, the IR spectra were recorded. ^1^HNMR spectra were acquired with a Bruker (Avance III) 400 MHz system spectrometer, with tetramethyl silane (TMS) serving as the internal standard and DMSO as the solvent. Chemical shifts were recorded in parts per million (ppm). The ^1^HNMR spectra notation included the symbols; singlet (s), doublet (d), triplet (t) and multiplet (m). Using the Electrospray Ionization (ESI) technique on a Bruker Fourier Transform Ion Cyclotron Resonance Mass Spectrometer, high-resolution mass spectra (HRMS) were produced.

### 4.2 Chemistry

Computationally screened analogs were synthesized (Scheme S1†) through three-step, esterification, hydrazide derivatives and coupling reaction between different aldehydes. The compounds were produced from the starting material (a) CH_3_OH, H_2_SO_4_ (b) NH_2_NH_2_·H_2_O, C_2_H_5_OH (c) RCHO, glacial CH_3_COOH by a series of reactions. The synthetic approach to the title compound is depicted in Scheme 1.^31^ In a 100 ml round-bottomed flask a mixture of 1 g (0.0028 mol) of indomethacin, 11.89 g (2.7 mol) of absolute methanol and 10 drops of concentrated sulphuric acid was placed, a reflux condenser was attached and the mixture was boiled gently for 5 hours. The rection was monitored by TLC with 1 : 4 ethyl acetate and petroleum ether. The excess alcohol was distilled off on a water bath (rotary evaporator) and allowed to cool. The residue was poured into a separating funnel and extraction was completed with ethyl acetate. Further, the organic layer was separated and the process was repeated for three times. The ethyl acetate layer was shaken with a strong solution of sodium hydrogen carbonate until all free acids were quenched and cessation of carbon dioxide gas. The organic layer was washed with water, and dried over anhydrous magnesium/sodium sulfate. The mixture was kept for half an hour with occasional shaking. The mixture was filtered and solvent was evaporated. The yellow sticky liquid of indomethacin methyl ester was collected and recrystallized from ethyl ether to recover the methyl ester as a yellow solid compound.^32^ Indomethacin methyl ester (0.01 mol, 3.71 gm) and hydrazine hydrate (purity 99%) (0.2 mol, 6.41 ml) were refluxed in absolute ethanol (50 ml) for 30 hours. The mixture was concentrated, cooled and poured on small quantity of crushed ice with stirring. The reaction mixture was kept at room temperature for 3 to 4 hours. The separated solid was filtered out, dried, and crystallized from ethanol. The solution of indomethacin hydrazide (1 mmol, 0.37 g) in ethanol (15 ml) containing appropriately substituted benzaldehydes (1.1 mmol) and a catalytic amount of glacial CH_3_COOH was refluxed for 3 hours. Further, the products were recovered by pouring the reaction mixture in ice cold water followed by filtration and washing with cold water. The products were recrystallized from ethanol and purified by column chromatography. Finally, ^1^H NMR, ^13^C NMR, and HRMS spectra were reported.

#### 4.2.1 (1-(4-Chlorobenzoyl)-5-methoxy-2-methyl-1*H*-indol-3-yl)-*N*′-(3-hydroxybenzylidene) aceto-hydrazide (IND-24)

Creamish compound, yield 81%; 108–110 °C; FTIR (KBr) cm^−1^: 3520–3413 (–OH, –NH), 3025–3091 (Ar), 1672 (C=O); ^1^H NMR (500 MHz, DMSO-*d*_6_) *δ* 8.23 (s, 1H), 7.68 (d, *J* = 7.5 Hz, 2H), 7.51 (d, *J* = 7.5 Hz, 2H), 7.33 (t, *J* = 7.5 Hz, 1H), 7.27–7.24 (m, 2H), 7.08 (t, *J* = 7.5, 1.5 Hz, 1H), 6.97 (dd, *J* = 7.5, 1.4 Hz, 1H), 6.90–6.88 (m, 2H), 6.73 (t, *J* = 1.4 Hz, 1H), 3.98 (s, 2H), 3.84 (s, 3H), 3.77 (s, 1H), 2.34 (s, 3H). ^13^C NMR (125 MHz, DMSO-*d*_6_) *δ* 171.80, 166.15, 157.09, 156.77, 147.91, 140.18, 137.73, 137.00, 134.75, 133.16, 130.69, 129.91, 128.89, 120.00, 119.36, 115.81, 114.72, 111.66, 111.47, 102.52, 56.03, 36.08, 11.31; MS (ESI): *m*/*z* found 476.137 [M^+^], 477.25 [M + 2], [M^+^: M + 2, 1 : 3]; calcd for C_26_H_22_ClN_3_O_4_: 475.13.

#### 4.2.2 (1-(4-Chlorobenzoyl)-5-methoxy-2-methyl-1*H*-indol-3-yl)-*N*′-(3-methoxybenzylidene) aceto-hydrazide (IND-25)

Greyish compound, yield 83%; mp 84–86 °C; FTIR (KBr) cm^−1^: 3372 (–NH), 3033–3089 (Ar), 1663 (C=O); ^1^H NMR (500 MHz, DMSO-*d*_6_) *δ* 9.87 (s, 1H), 7.67–7.62 (m, 3H), 7.46–7.44 (m, 5H), 7.36–7.31 (m, 12H), 7.21–7.19 (m, 2H), 7.08–7.05 (m, 3H), 6.87 (t, *J* = 1.5 Hz, 1H), 4.24 (s, 2H), 3.84 (s, 3H), 3.82 (s, 3H), 2.49 (s, 3H). ^13^C NMR (125 MHz, DMSO-*d*_6_) *δ* 171.80, 166.15, 160.29, 157.09, 147.91, 140.18, 137.73, 137.03, 134.75, 133.16, 129.91, 129.20, 128.89, 121.23, 115.81, 114.88, 112.19, 111.66, 111.47, 102.52, 56.03, 36.08, 11.22.; MS (ESI): *m*/*z* found 490.28 [M^+^], 491.36 [M + 2], [M^+^: M + 2, 1 : 3]; calcd for C_27_H_24_ClN_3_O_4_:489.96.

#### 4.2.3 (1-(4-Chlorobenzoyl)-5-methoxy-2-methyl-1*H*-indol-3-yl)-*N*′-(4-hydroxybenzylidene)aceto-hydrazide (IND-30)

Light yellow compound, yield 84%; mp 106–110 °C; FTIR (KBr) cm^−1^: 3472–3405 (–OH, –NH), 3020–3085 (Ar), 1681 (C=O); ^1^H NMR (500 MHz, DMSO-*d*_6_) *δ* 8.58 (s, 1H), 7.78 (d, *J* = 7.5 Hz, 2H), 7.64 (s, 1H), 7.52 (d, *J* = 7.5 Hz, 2H), 7.20 (d, *J* = 7.5 Hz, 1H), 7.00 (t, *J* = 16.3, 7.5 Hz, 5H), 6.76 (d, *J* = 1.6 Hz, 1H), 4.15 (s, 2H), 3.81 (s, 3H), 2.58 (s, 1H), 2.30 (s, 3H). ^13^C NMR (125 MHz, DMSO-*d*_6_) *δ* 171.80, 166.15, 158.32, 157.09, 148.64, 140.18, 137.73, 134.75, 133.16, 129.91, 129.72, 128.89, 126.92, 115.81, 115.59, 111.66, 111.47, 102.52, 56.03, 36.08, 10.97; MS (ESI): *m*/*z* found 476.137 [M^+^], 477.25 [M + 2], [M^+^: M + 2, 1 : 3]; calculated for C_26_H_22_ClN_3_O_4_: 475.93.

#### 4.2.4 (1-(4-Chlorobenzoyl)-5-methoxy-2-methyl-1*H*-indol-3-yl)-*N*′-(2-methoxybenzylidene)aceto-hydrazide (IND-31)

Gray colored compound, yield 77%; mp 80–82 °C; FTIR (KBr) cm^−1^: 3367 (–NH), 3041–3091 (Ar), 1654 (C=O); ^1^H NMR (500 MHz, DMSO-*d*_6_) *δ* 9.86 (s, 1H), 7.68 (d, *J* = 7.5 Hz, 2H), 7.44 (d, *J* = 7.5 Hz, 2H), 7.36–7.34 (m, 3H), 7.17 (t, *J* = 20.7, 7.4, 1.4 Hz, 3H), 7.06 (dd, *J* = 7.5, 1.4 Hz, 1H), 6.90 (d, *J* = 1.4 Hz, 1H), 4.25 (d, *J* = 17.4 Hz, 6H), 3.05 (s, 2H), 2.49 (s, 3H). ^13^C NMR (125 MHz, DMSO-*d*_6_) *δ* 171.80, 166.15, 159.58, 157.09, 149.82, 140.18, 137.73, 134.75, 133.16, 129.91, 128.89, 128.33, 127.89, 125.51, 121.54, 115.81, 113.69, 111.66, 111.47, 102.52, 56.78, 56.03, 36.08, 11.33; MS (ESI): *m*/*z* found 490.63 [M^+^], 491.32 [M + 2], [M^+^: M + 2, 1 : 3]; calculated for C_27_H_24_ClN_3_O_4_: 489.96.

#### 4.2.5 (1-(4-Chlorobenzoyl)-5-methoxy-2-methyl-1*H*-indol-3-yl)-*N*′-(2,6-dihydroxybenzylidene)aceto-hydrazide (IND-40)

Light yellow colored compound, yield 77%; mp 104–106 °C; FTIR (KBr) cm^−1^: 3475–3390 (–OH, –NH), 3025–3091 (Ar), 1678 (C=O); ^1^H NMR (500 MHz, DMSO-*d*_6_) *δ* 9.76 (s, 1H), 7.67 (d, *J* = 7.5 Hz, 2H), 7.44 (d, *J* = 7.5 Hz, 2H), 7.41 (s, 1H), 7.37 (d, *J* = 7.5 Hz, 1H), 7.35 (t, *J* = 7.5 Hz, 1H), 6.92 (dd, *J* = 7.5, 1.4 Hz, 1H), 6.86 (d, *J* = 1.4 Hz, 1H), 6.44 (d, *J* = 7.5 Hz, 2H), 4.29 (s, 2H), 3.85 (s, 3H), 3.58 (s, 2H), 2.49 (s, 3H). ^13^C NMR (125 MHz, DMSO-*d*_6_) *δ* 171.80, 166.15, 159.69, 157.09, 143.26, 140.18, 137.73, 134.75, 133.16, 131.97, 129.91, 128.89, 115.81, 112.56, 111.66, 111.47, 109.24, 102.52, 56.03, 36.08, 10.87.; MS (ESI): *m*/*z* found 492.17 [M^+^], 493.77 [M + 2], [M^+^: M + 2, 1 : 3]; calculated for C_26_H_22_ClN_3_O_5_: 491.93.

#### 4.2.6 (1-(4-Chlorobenzoyl)-5-methoxy-2-methyl-1*H*-indol-3-yl)-*N*′-(2,3-dihydroxybenzylidene)aceto-hydrazide (IND-42)

Yellowish colored compound, yield 75%; mp 108–110 °C; FTIR (KBr) cm^−1^: 3481–3392 (–OH, –NH), 3018–3083 (Ar), 1684 (C=O); ^1^H NMR (500 MHz, DMSO-*d*_6_) *δ* 8.37 (s, 1H), 7.67 (s, 1H), 7.66 (d, *J* = 7.5 Hz, 2H), 7.50 (s, 1H), 7.45 (d, *J* = 7.5 Hz, 2H), 6.90 (d, *J* = 7.5 Hz, 1H), 6.89 (dd, *J* = 7.5, 1.6 Hz, 1H), 6.56 (dd, *J* = 27.2, 16.8, 7.2 Hz, 4H), 4.08 (s, 2H), 3.84 (s, 3H), 3.43 (s, 1H), 2.83 (s, 1H), 2.39 (s, 3H). ^13^C NMR (125 MHz, DMSO-*d*_6_) *δ* 171.80, 166.15, 157.09, 149.42, 146.94, 144.69, 140.18, 137.73, 134.75, 133.16, 129.91, 128.89, 122.00, 121.33, 119.89, 115.81, 111.66, 111.47, 102.52, 56.03, 36.08, 11.41.; MS (ESI): *m*/*z* found 492.19 [M^+^], 493.73 [M + 2], [M^+^: M + 2, 1 : 3]; calculated for C_26_H_22_ClN_3_O_5_: 491.93.

### 4.3 *In vitro* study

#### 4.3.1 AChE and BuChE inhibition assays

With minor modifications, Ellman *et al.*, 1961's method for AChE and BuChE inhibition experiments was employed.^32^ AChE and BuChE were provided by Sigma Aldrich (CAS No. 9000-81-1, 9001-08-5 respectively). Himedia supplied Acetylthiocholine iodide (AthCI), Butyrylthiocholine iodide (BthCI), and 5,5′-dithiobis (2-nitrobenzoic acid) (DTNB-reagent). The assays were carried out in a tris-HCl buffer (pH 8.0) with donepezil serving as the reference standard drug. Moreover, ten different doses of test substances were used for IC_50_ assessment (1000 μM, 500 μM, 250 μM, 100 μM, 50 μM, 25 μM, 10 μM, 1 μM, 0.1 μM and 0.01 μM). AChE (50 μL, 1.00 U mL^−1^) or BuChE (50 μL, 0.6 U mL^−1^) and 20 μL of the test or standard compounds were incubated in a 96 well plate for 30 minutes at room temperature. The aforesaid solution was supplemented with 100 μL (1.5 mM) of DTNB. The substrate, acetylthiocholine iodide (15 mM, 10 μL) or butyrylthiocholine iodide (30 mM, 10 μL), was added and immediately absorbance was measured at 415 nm for 20 minutes at intervals of one minute using a Synergy HTX multi-mode reader (BioTek, USA). The IC_50_ values were determined by measuring the absorbance of the test and standard substances. Three independent runs of the assays were conducted in triplicate.

#### 4.3.2 Cell line-based toxicity study on SH-SY5Y cell line

The neuroprotection property of selected compound IND-30 was evaluated against SH-SY5Y neuroblastoma cell lines by the MTT assay according to the literature procedure with minor modifications.^33^ Briefly, the cell lines (density 1 × 10^4^ cells per wells) were plated in 96 well plates and incubated for 24 h at 37 °C in a humidified atmosphere with 5% CO_2_. The test compounds in different concentration ranges (compound IND-30 – 10, 20, 30, 40 and 50 mM) were added, and cells were incubated for 72 h. Further, 20 μL/well of MTT reagent was added, and the cells were incubated for an additional 2 h. The obtained purple-coloured formazan crystals were solubilized in a dissolving solvent (100 μL/well DMSO). The absorbance was measured at 570 nm, and the percent cell viability was calculated. Each treatment was executed in triplicate and data were presented as a percentage of the control.

#### 4.3.3 AChE-induced Aβ_1–42_ aggregation assay

Reactive oxygen species (ROS) was found to be produced by Aβ-redox active metal complex by a Fenton-type reaction, which induced oxidative stress leading to the death of neurons. The Aβ oligomers and aggregates formed with or without AChE and metal ions induce multifaceted toxicity leading to neuronal cell death. Hence, a multifaceted approach is needed to overcome the toxicity produced by Aβ aggregates. The assay was performed to determine the inhibition potential of compound IND-30 against AChE induced Aβ_1–42_ aggregation.^34,35^ Aβ_1–42_, purchased from Sigma, was dissolved in 10 mM phosphate buffer (PBS) of pH 7.5 to get a stock concentration of 200 mM. The test compounds were dissolved in the DMSO and further diluted with PBS. Different proportions of the Aβ_1-42_: Inhibitor (1 : 0.05, 1 : 1, 1 : 2) were used in the ThT assay. The final concentration of Aβ_1–42_, compound IND-30 and AChE was 10 μM (2 μL), 0.5, 10, 20 μM (2 μL) and 230 μM (16 μL) respectively. The mixtures were incubated at room temperature for 48 hours in the dark. The fluorescence intensities of the incubated mixtures were measured by adding 178 μL of 20 μM thioflavin T (ThT) at excitation and emission wavelengths of 485 and 528 nm The ThT assay was performed in triplicate and three independent runs.

### 4.4 Molecular docking

The X-ray crystal structure of AChE complexed with donepezil (E2020) was downloaded from the online Protein Data Bank (PDB code: 1EVE, resolution: 2.5, http://www.rcsb.org/). A and B chains make up the 543 amino acids that make up the 1EVE protein.^36^ Standard donepezil was coupled with Chain A, which was chosen for *in silico* investigations. The protein for AutoDockVina was created in accordance with our previously published research report.^30^ Selected ligands including the standard donepezil (PDB: E2020) were put through the Pyrx 0.8 virtual screening tool. This was done utilizing the AutoDock Vina wizard platform. Both macromolecules and ligands were transformed into pdbqt for docking. The centroid grid box surrounding the *x*, *y*, and *z* coordinates of TcAChE was established for blind docking (73.57 × 73.93 × 65.85). The grid was modified to include only the interacting amino acids of 1EVE: TRP A: 84, PHE A: 330, PHE A: 331, TYR A: 334, TRP A: 279, LEU A: 282, and TYR A: 70 around the *x*, *y*, and *z* coordinates of the 1EVE-prep macromolecule [31.18 × 34.24 × 25.22]. This was done to validate blind docking. Biovia Discovery Studio (BDS) 4.5 was used to evaluate the docking output files (http://www.3dsbiovia.com/). According to Kryger *et al.*,^37^ the phenyl ring of E2020 was generated by stacking with Trp84 and Phe330 and another aromatic ring with Trp-279 was a phenyl ring. A hydrogen bond was additionally seen between Phe-288 and the ketone oxygen.^19^

### 4.5 Molecular dynamics simulations (MDSs)

Simulations of molecular dynamics were done with the Gromacs 2020.4 version. The topology files for the IND-24, IND-30, IND-40, and IND-42 ligands and the acetylcholinesterase enzyme were made with CgenFF and the Charmm36-Jul2020 force field for the pdb2gmx script, respectively.^38,39^ The MDS was done with Berendsen's coupling algorithms, V-rescale, and the Newtonian leap-frog MD integrator. The boundary conditions were changed periodically. When nine Na^+^ ions and a TIP3P water molecule were added to systems 47 and 48 to neutralize it, the solvating was made. The energy was minimized by the protein–ligand isothermal-isobaric (amount of substance, *N*, volume, *V*, and equilibrium steps temperature, *T* (*NVT*)) and canonical ensemble (amount of substance, *N*, pressure, *P*, and temperature, *T* (*NPT*) were carried out at 300 K and 1 atm for 100 ps. The rvind ions of dynamics were run for 30 ns. Root means square deviation (RMSD), root mean square fluctuation (RMSF), and analyses of hydrogen bonds between molecules were used to measure the relationship between acetylcholinesterase enzyme^40^ and IND-24, IND-30, IND-40, and IND-42 ligands. The QtGrace tool was used to make graphs of the paths of dynamic simulations.^41^

### 4.6 DFT/B3LYP calculations

All of the quantum chemical properties of all substances were calculated with the Gaussian 09 package program^42^ and the Density Functional Theory (DFT/B3LYP/6-311G (d,p)) method. Based on HOMO–LUMO energies of compounds, Δ*E* (*E*_LUMO_ – *E*_HOMO_), ionization potential (IP = −HOMO), electron affinity (EA = −LUMO), electronegativity (*X* = [IP + EA]/2), chemical hardness (*η’* = [IP − EA]/2), chemical softness (*S* = 1/2*η’*), chemical potential (*μ* = −[IP + EA]/2) and electrophilic index (*ω* = μ2/2*η*) were calculated. Also, Theoretical results for molecular electrostatic potential (MEP) of compounds were derived.^43^ The GaussView 6.0.16 program^44^ was used to visualize the results. Celik *et al.* used the B3LYP technique to conduct DFT investigations against AChE, BuChE, and tyrosinase, which are important targets in Alzheimer's disease.

### 4.7 Lead compounds selection criteria

The final lead compounds were selected utilizing two sets of criteria spanning two stages of the process, including protein–ligand binding affinity, drug-like characteristics, and ligand-target protein physiochemical interactions. The blind docking phase one selection criteria were utilized to choose ligands having a docking score of less than −11 kcal mol^−1^. In the second step of candidate compound selection, two parameters are taken into account. The first was the ability to interact with amino acids involved in acceptor substrate placement, and the second was drug-like qualities evaluated by Lipinski's rule of five, Veber's rules criteria, Muegge's rules, and a toxicities study. These filtration criteria would lead us to conclude that prospective MDS chemicals and AD medication design exist.

## Abbreviations

AD: Alzheimer's disease
A*β*: Amyloid-beta
MDS: Molecular dynamic simulation
AchE: Acetylcholine esterase
HOMO: Highest occupied molecular orbital
LUMO: Lowest unoccupied molecular orbital
DFT: Density functional theory
PDB: Protein data bank

## Conflicts of interest

There are no conflicts to declare.

## Supplementary Material

RA-013-D3RA03224H-s001
