# Supplementary material for: Development of substituted benzylidene derivatives as novel dual cholinesterase inhibitors for Alzheimer's treatment
Source: RSC Adv. 2023 Sep 4;13(38):26344–56. doi: 10.1039/d3ra03224h (PMC10476022; doi:10.1039/d3ra03224h)
Supplement: RA-013-D3RA03224H-s001 [file RA-013-D3RA03224H-s001.pdf]

Electronic Supplementary Material (ESI) for RSC Advances.
This journal is © The Royal Society of Chemistry 2023

Development of substituted benzylidene derivatives as Novel Dual Cholinesterase Inhibitors for Alzheimer's treatment

Shraddha Manish Gupta[1,2], Ashok Behera[3], Neetesh K Jain[1], Avanish Tripathi[4], Dinesh Rishipathak[5], Siddharth Singh[2], Nafees ahemad[6], Meryem Erol[7], Devendra Kumar[8*]

[1]Faculty of Pharmacy, Oriental University, Indore-453555, Madhya Pradesh, India.

[2]Department of Pharmaceutical Sciences, School of Health Sciences and Technology, University of Petroleum and Energy Studies (UPES), Dehradun- 248007, India.

[3]Faculty of Pharmacy, School of pharmacy and population health informatics, DIT University, Makkawala, Dehradun, Uttarakhand, India.

[4]Institute of Pharmaceutical Research, GLA University, Mathura-281 406 (U.P.), India.

[5]Department of Pharmaceutical Chemistry, MET's Institute of Pharmacy, Nasik, Maharashtra, India

[6]School of Pharmacy, Monash University, Jalan lagoon selatan, Bandar Sunway, Petaling Jaya 47500, Selangor DE, Malaysia.

[7]Department of Pharmaceutical Chemistry, Faculty of Pharmacy, Erciyes University, Kayseri, Turkey

[8]School of Pharmacy & Technology Management, SVKM's NMIMS (Deemed-to-be) University, Mukesh Patel Technology Park, Shirpur 425405, India.

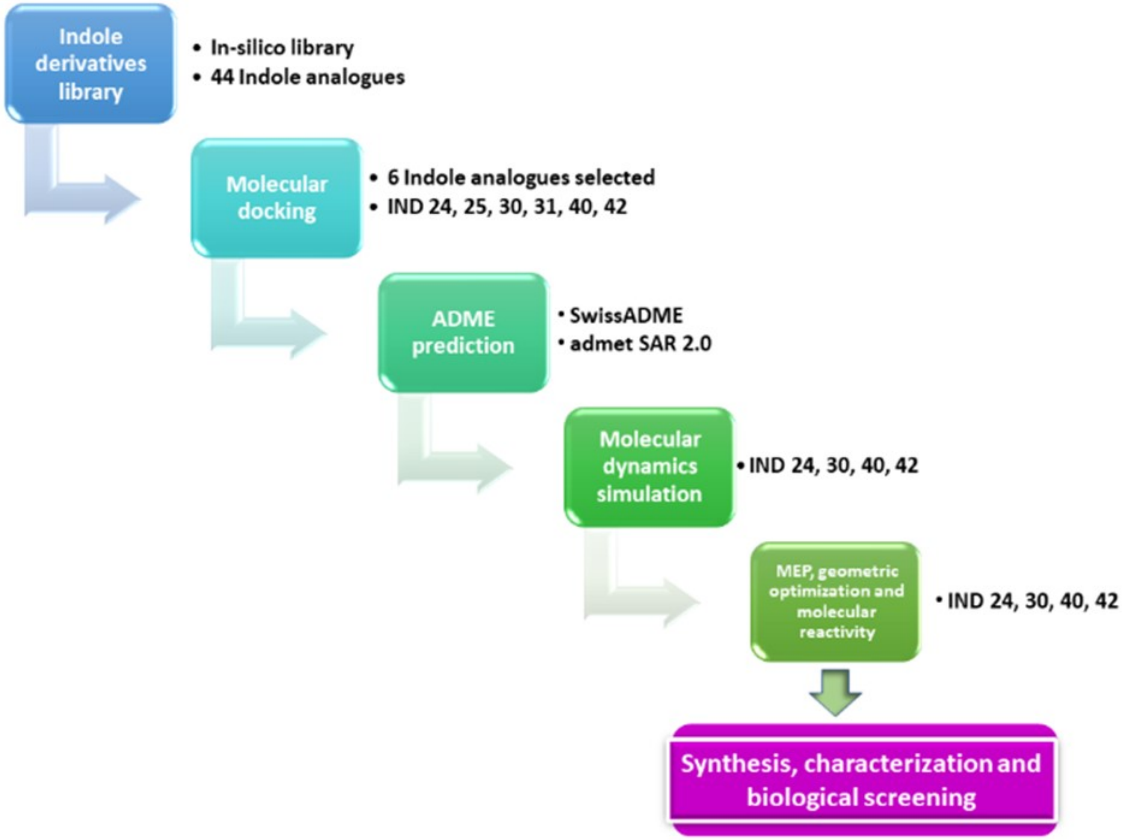

Figure S1. Procedure and rationale of designing Indole analogs followed by synthesis, characterization, and biological screening**.**

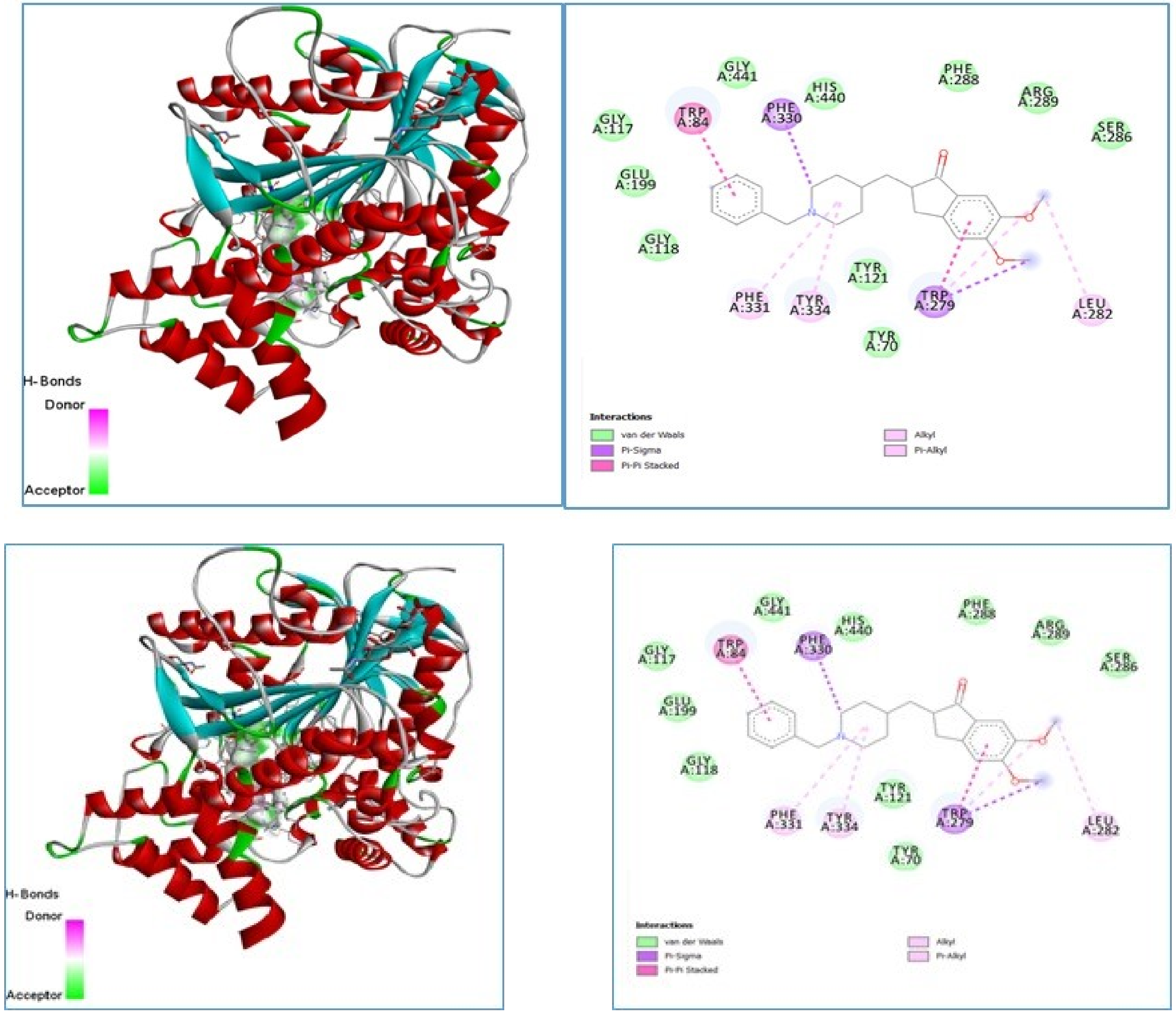

figure S2. Blind docking poses for the validation of docking

Spectral data of synthesized compounds

^{H}NMR data

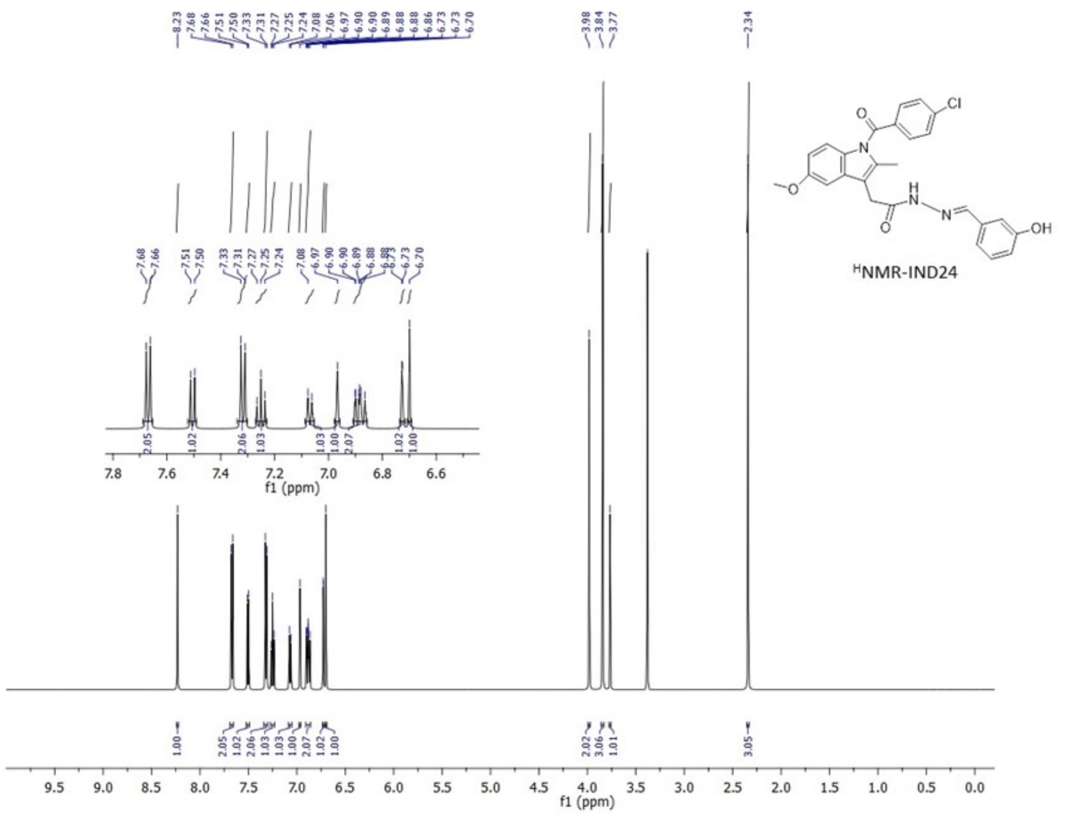

Figure S3. ^{1}H NMR Spectra of IND-24

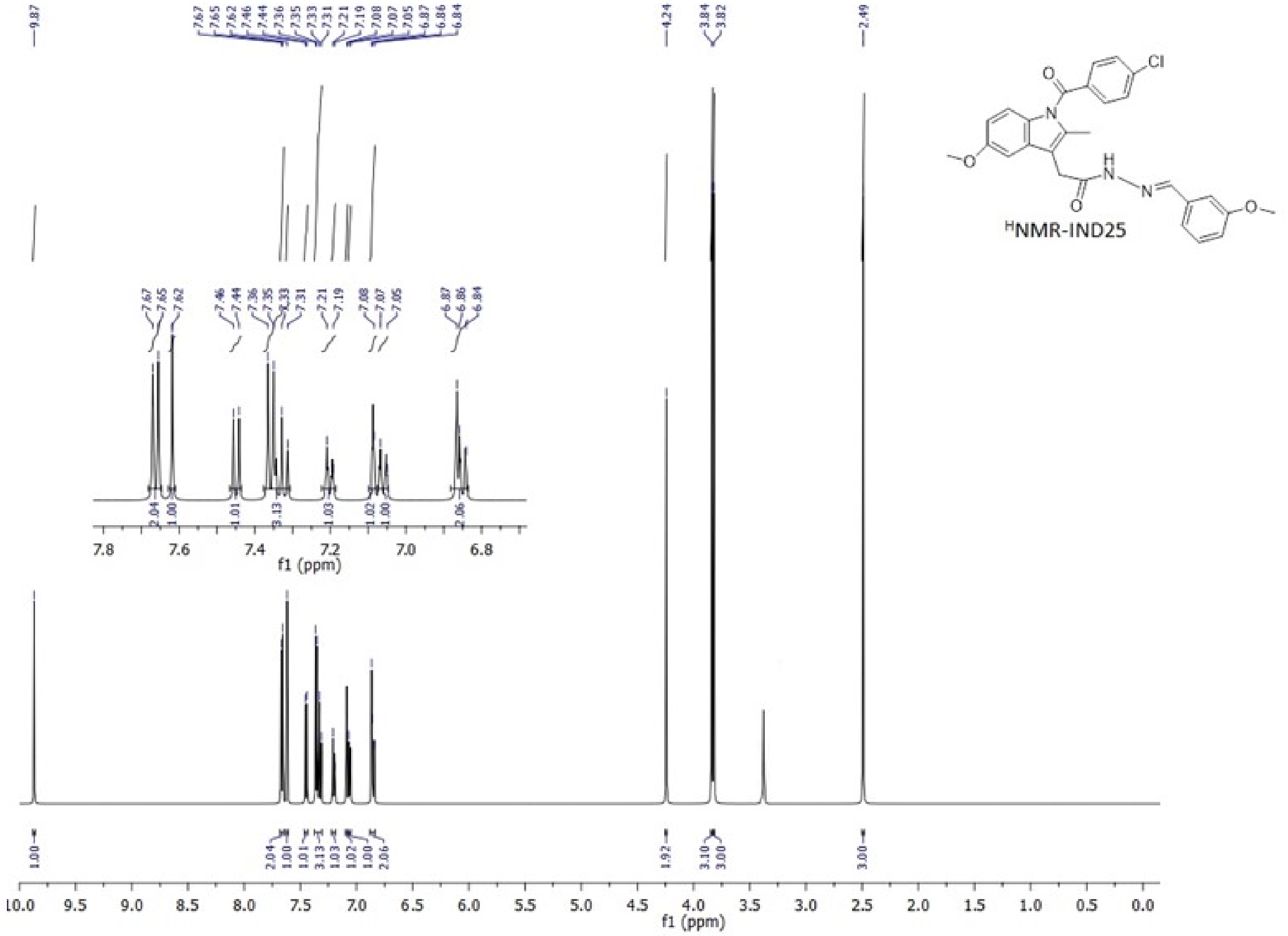

Figure S4. ¹H NMR Spectra of IND-25

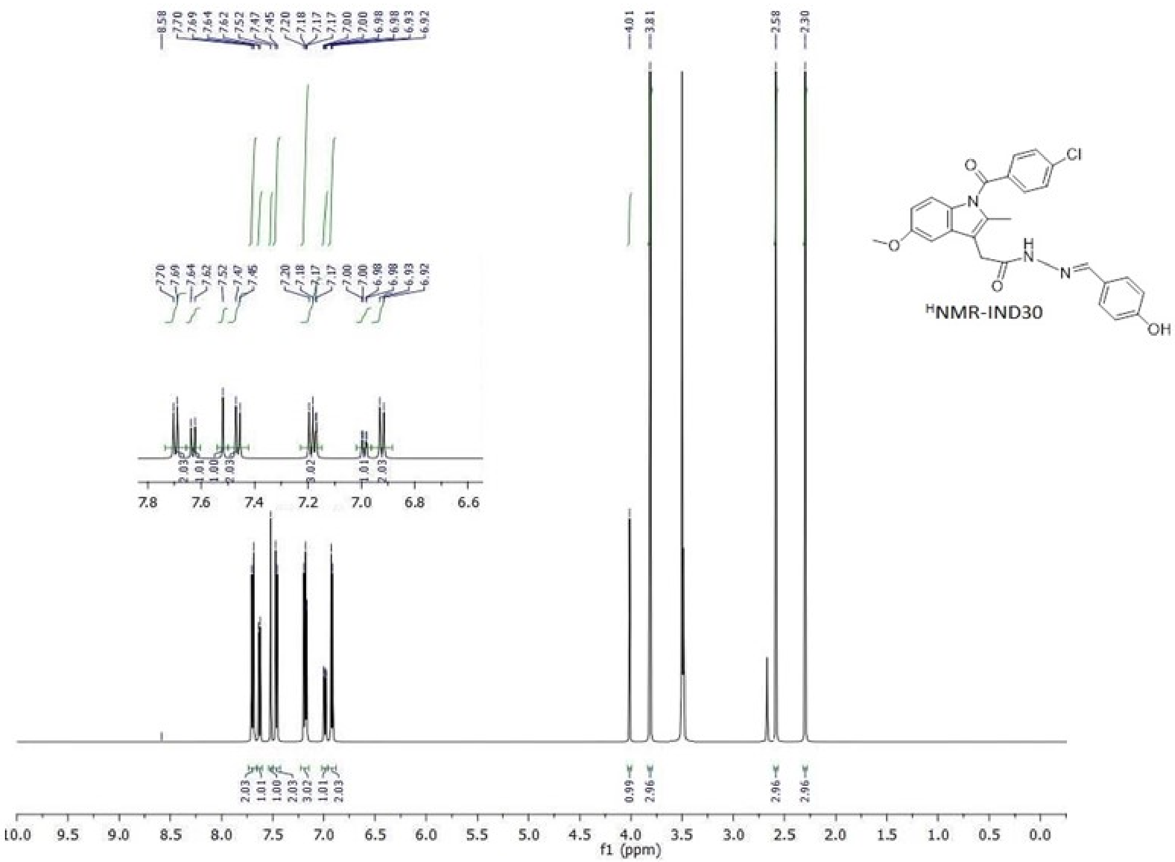

Figure S5. ^{1}H NMR Spectra of IND-30

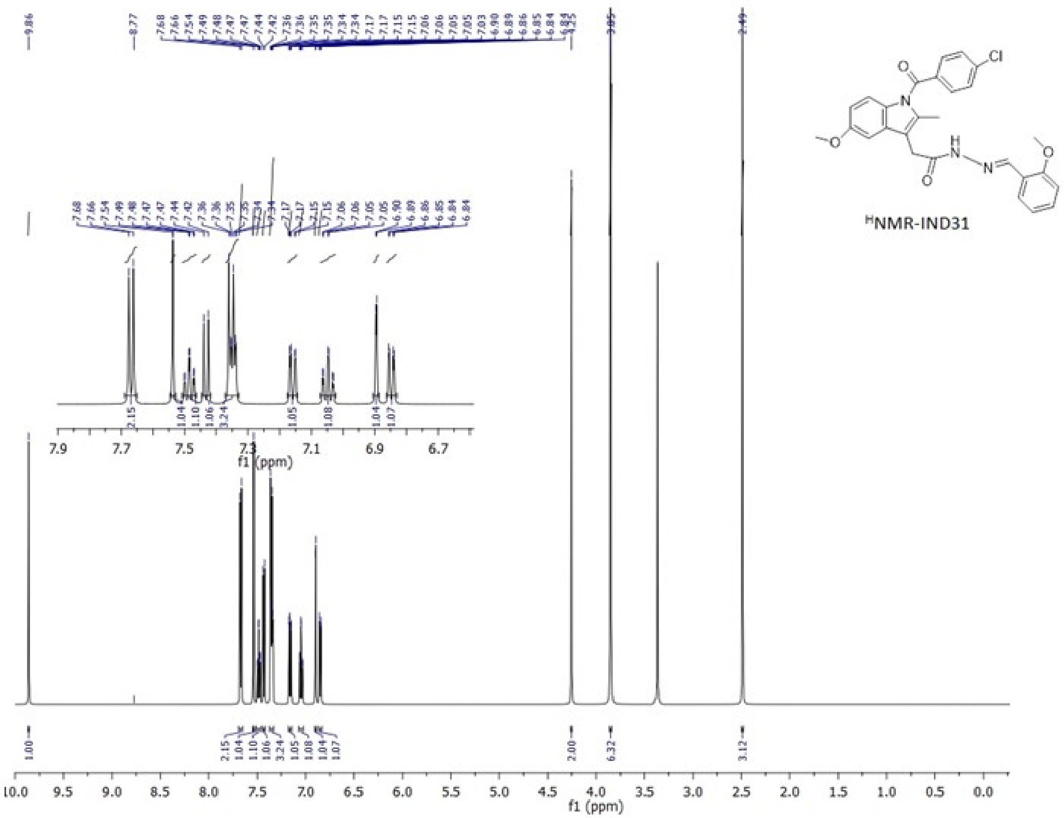

Figure S6. ^{1}H NMR Spectra of IND-31

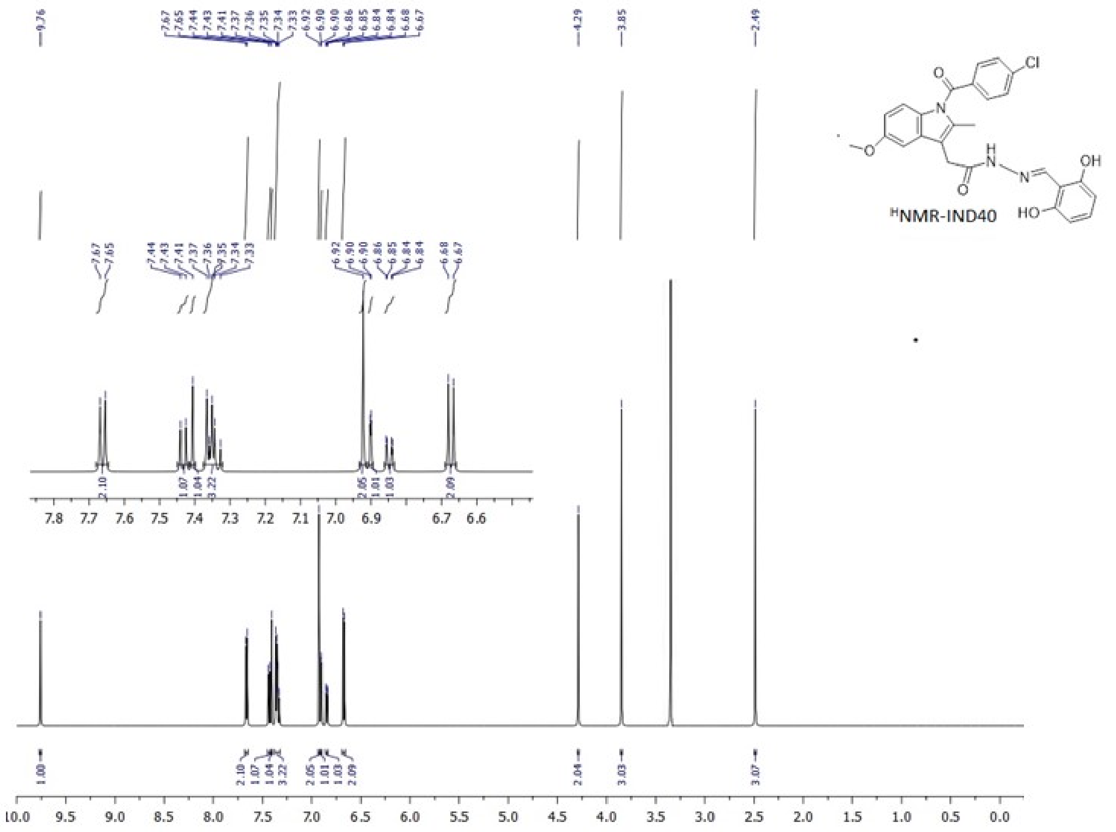

Figure S7. ^{1}H NMR Spectra of IND-40

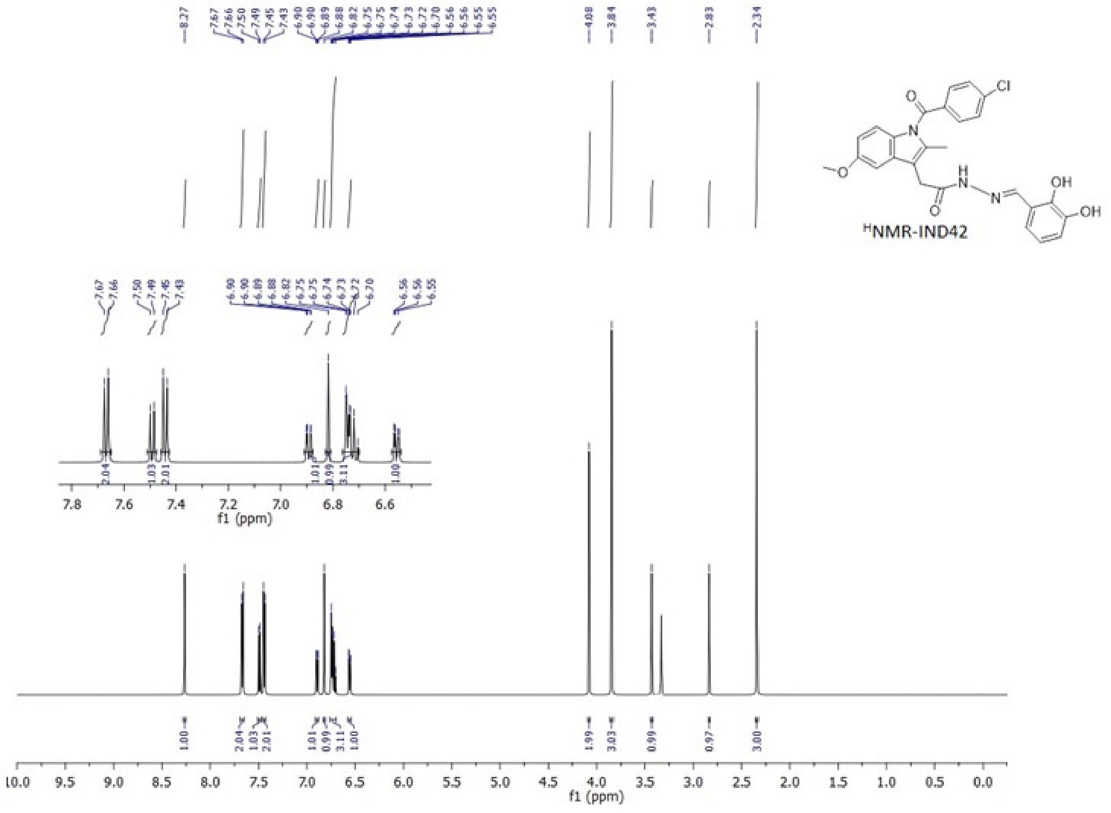

Figure S8. ^{1}H NMR Spectra of IND-42

CNMR data

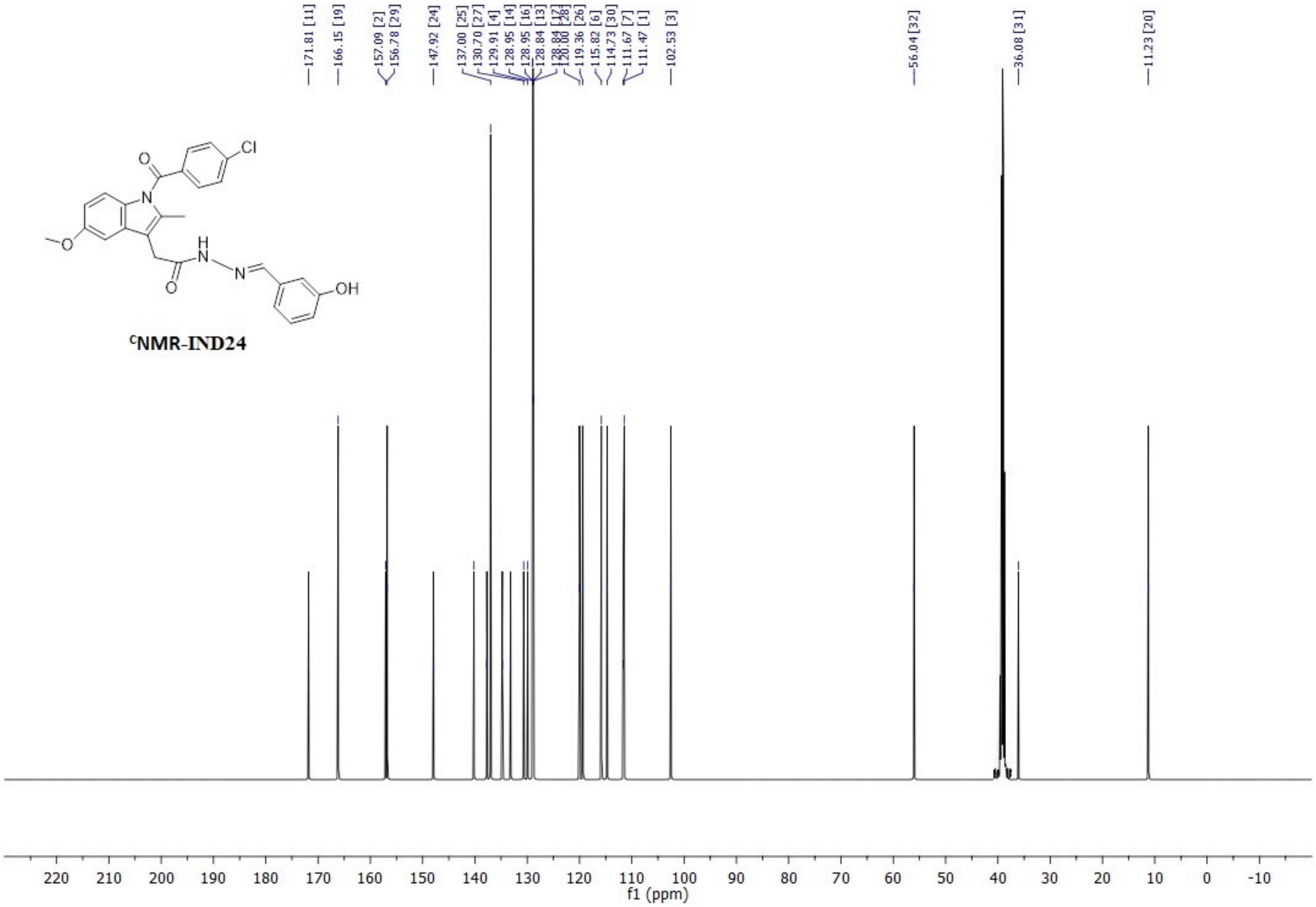

Figure S9. ^{13}C NMR Spectra of IND-24

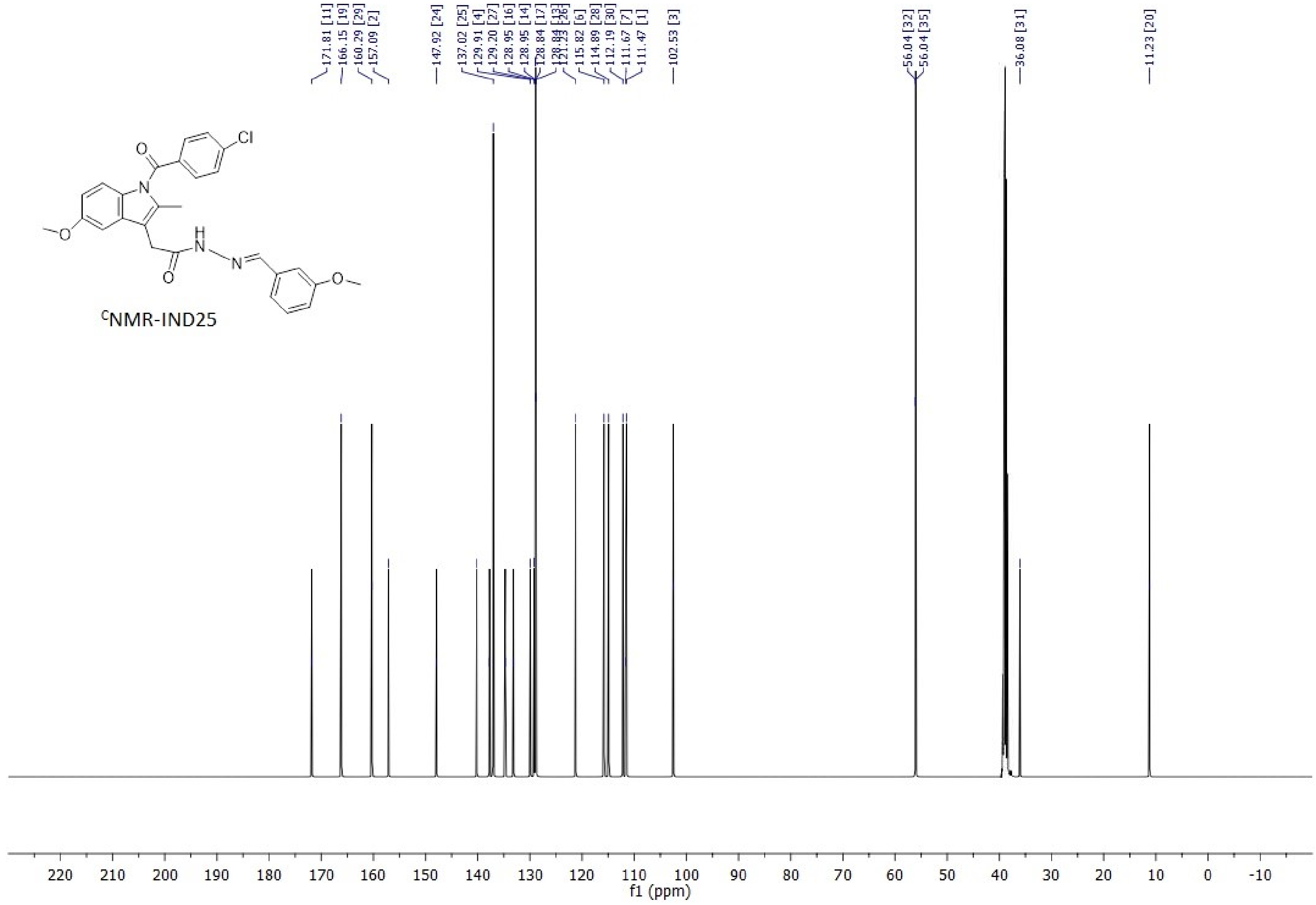

Figure S10. ^{13}C NMR Spectra of IND-25

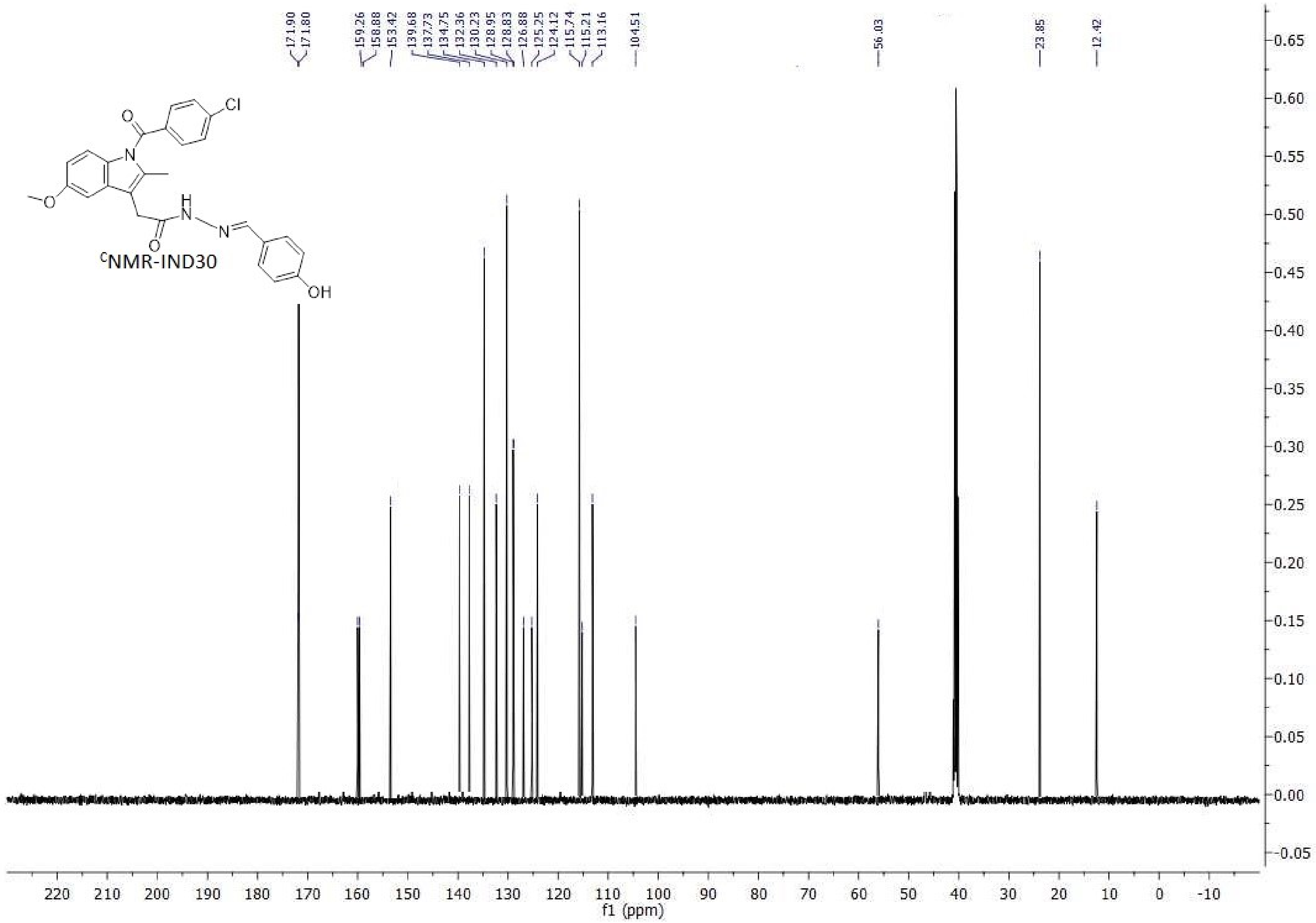

Figure S11. ^{13}C NMR Spectra of IND-30

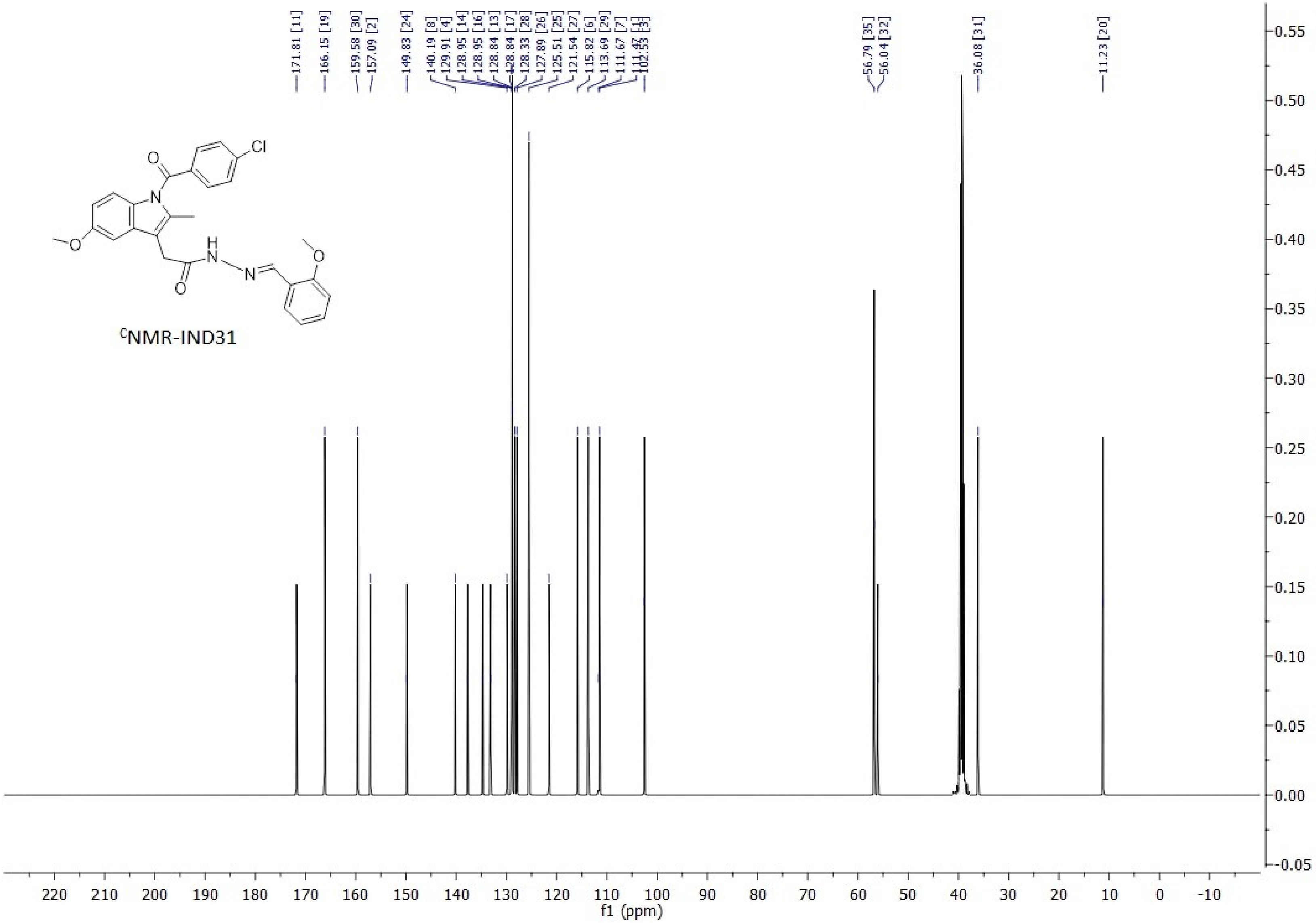

Figure S12. ^{13}C NMR Spectra of IND-31

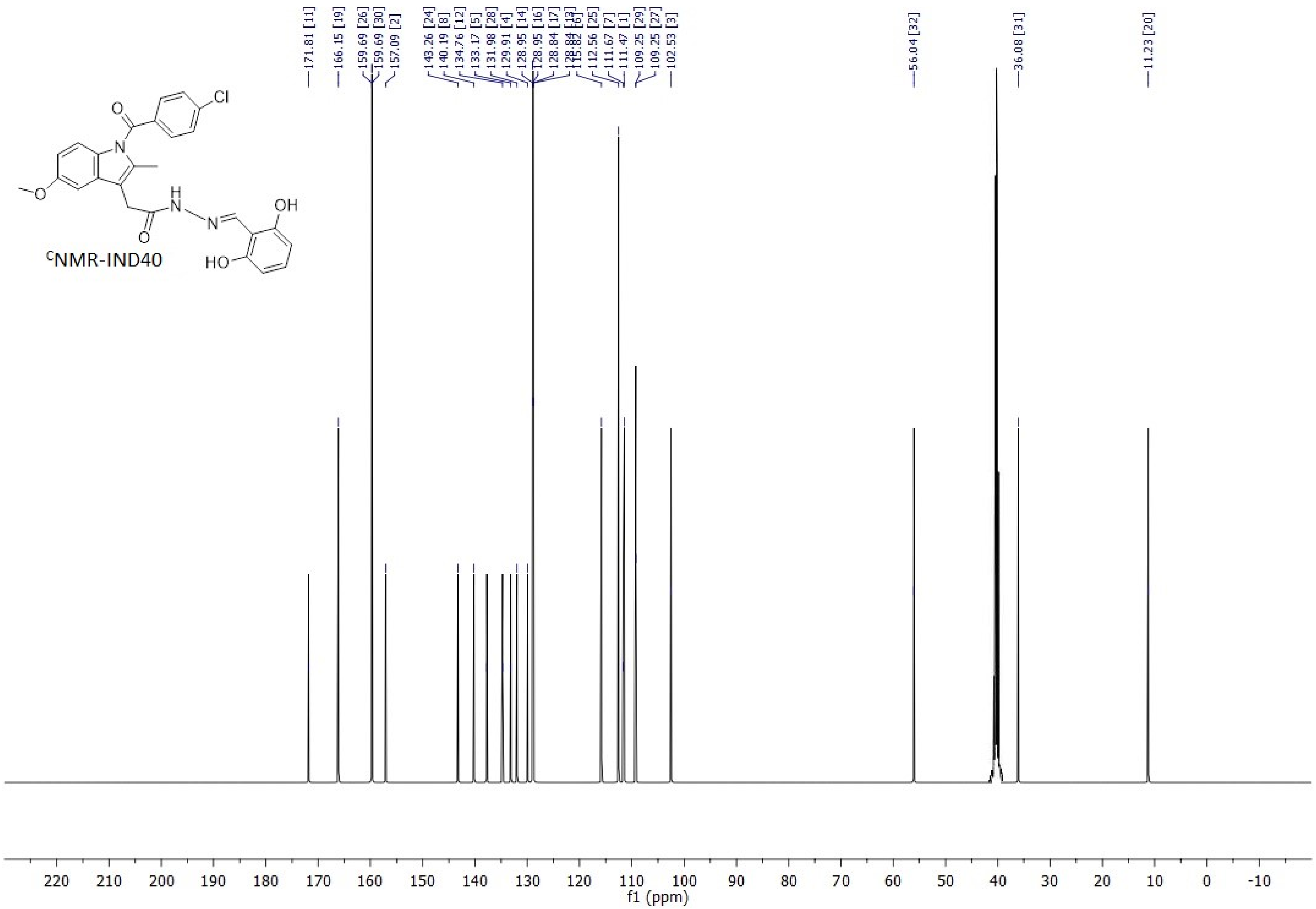

Figure S13. ^{13}C NMR Spectra of IND-40

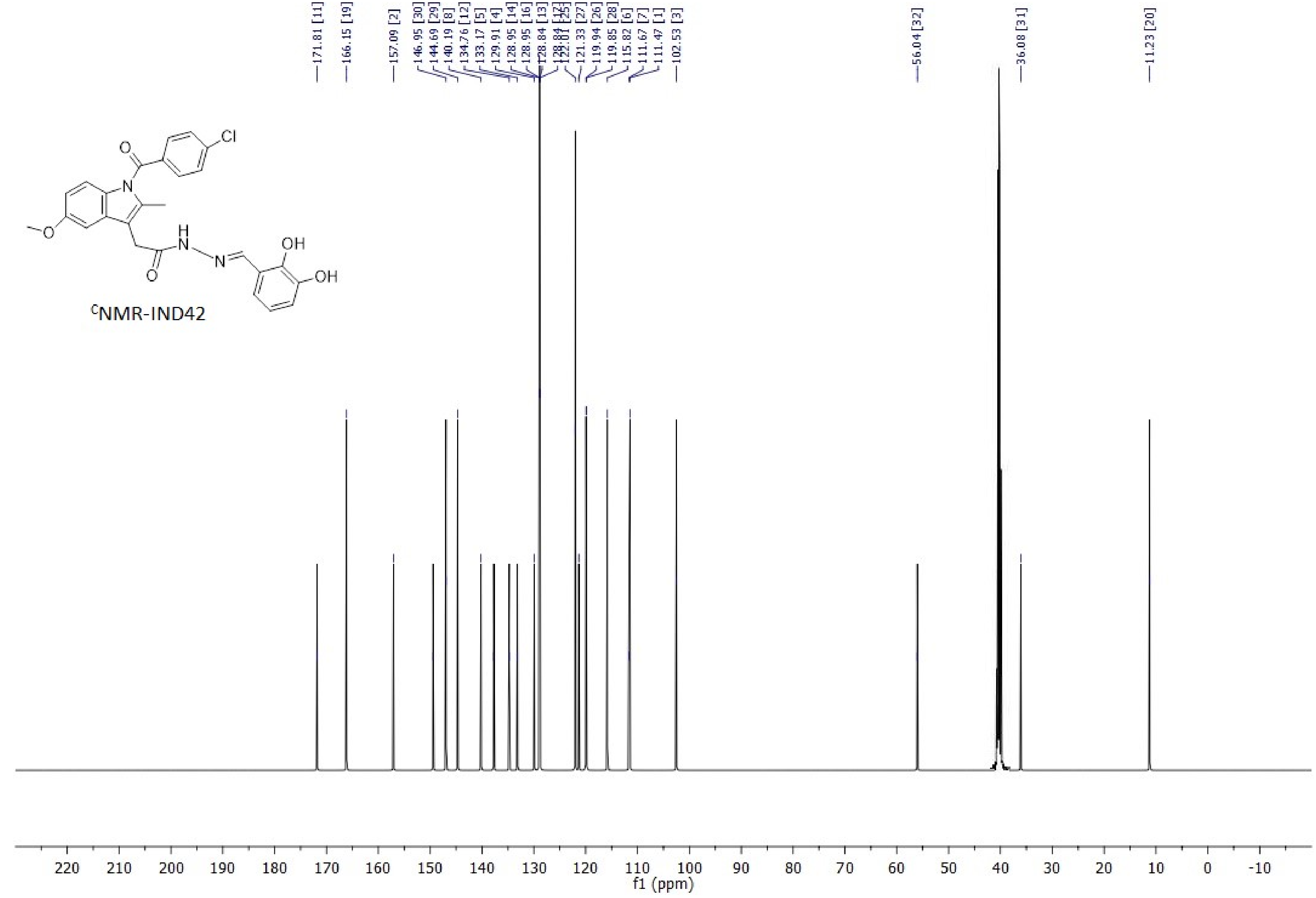

Figure S14. ^{13}C NMR Spectra of IND-42

Mass Spectra

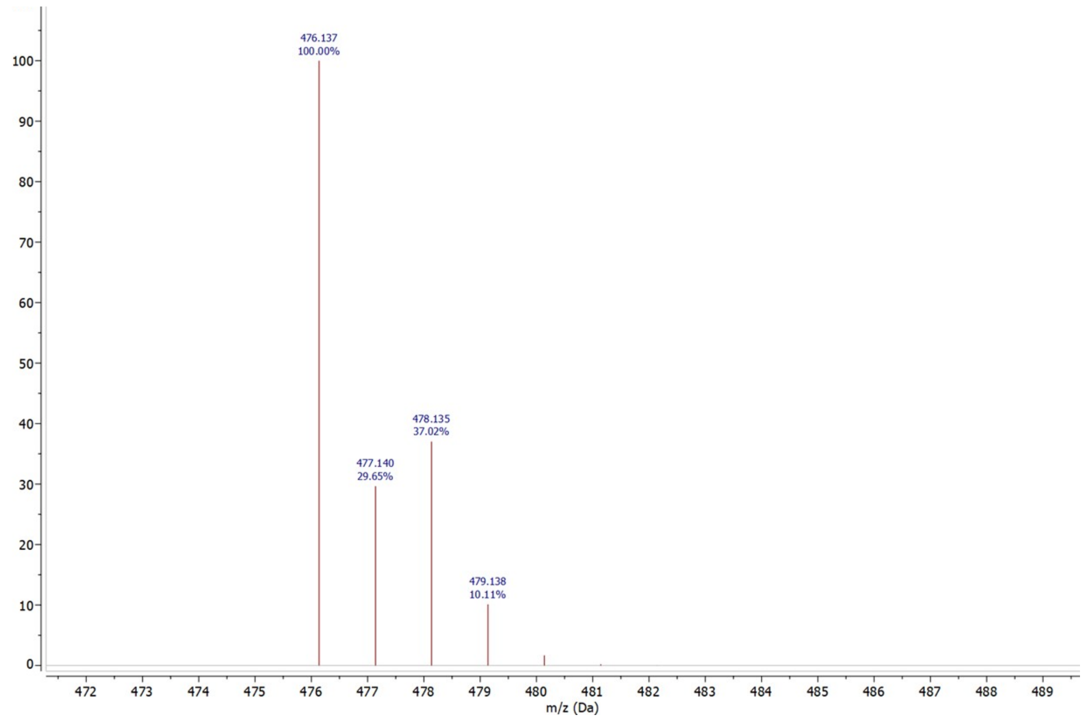

Figure S15. Mass Spectra of IND-24

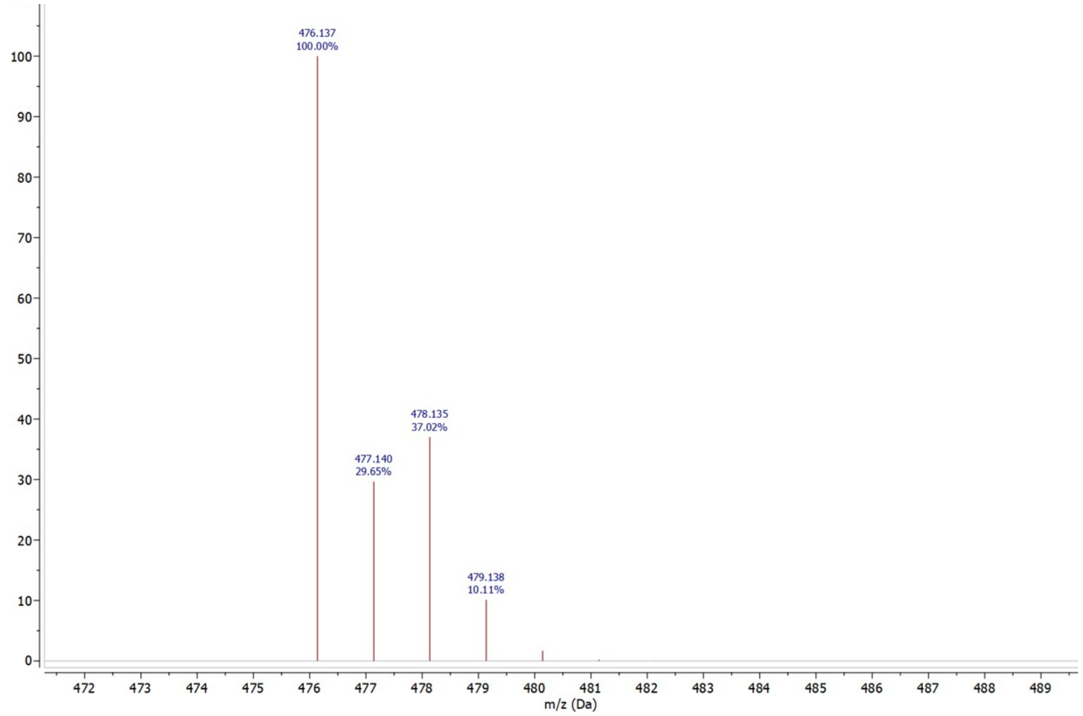

Figure S16. Mass Spectra of IND-30

Table S1. Docking Results of 44 indomethacin derivatives.

Table S2. The physiochemical, lipophilicity, water solubility, pharmacokinetics, drug likeliness and toxicity predictions of 44 indomethacin derivatives.

Table S1. Docking Results of 44 indole derivatives.

Ligand Code	Structure of Ligand	Binding Energy (kcal/Mol)
		AChE (1eve) docking
**IND**	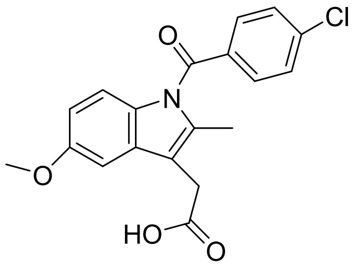	-10.1
**IND-1**	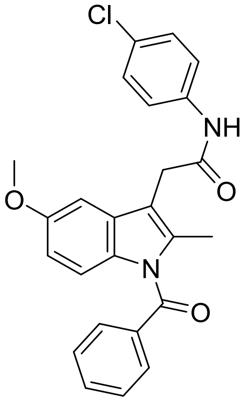	-11.0

**IND-2**	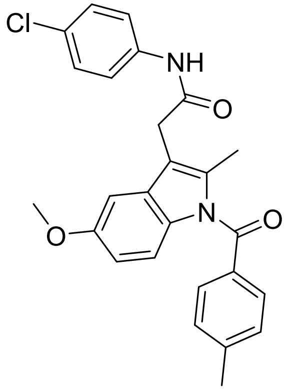	-11.2
**IND-3**	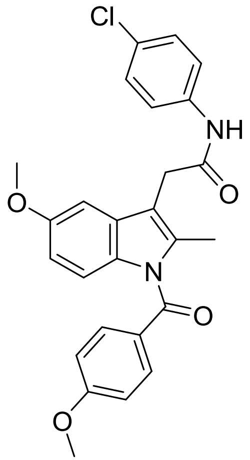	-11.4

**IND-4**	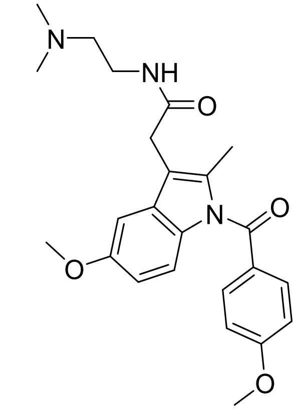	-10.2
**IND-5**	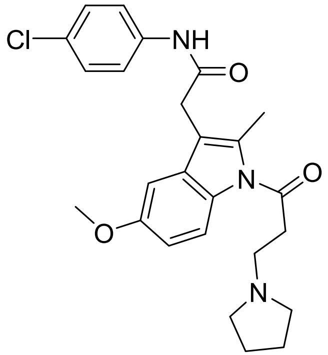	-10.2
**IND-6**	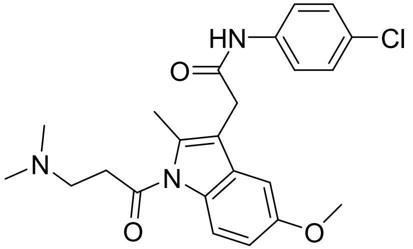	-9.2

**IND-7**	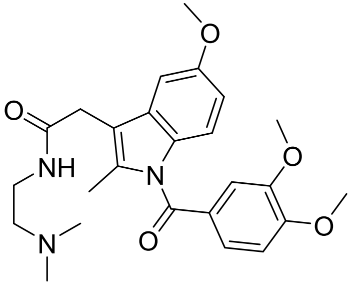	-9.0
**IND-8**	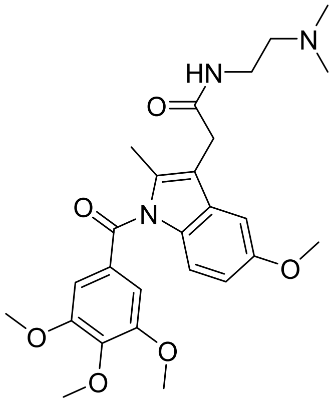	-8.1
**IND-9**	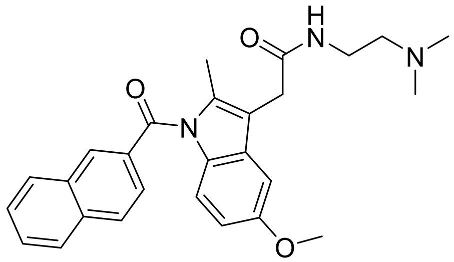	-11.1

**IND-10**	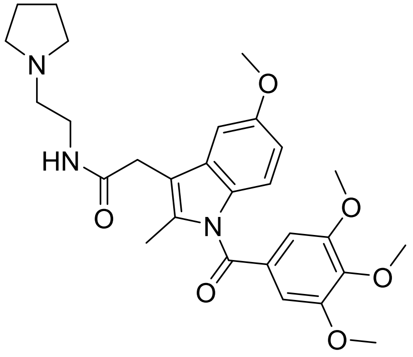	-8.8
**IND-11**	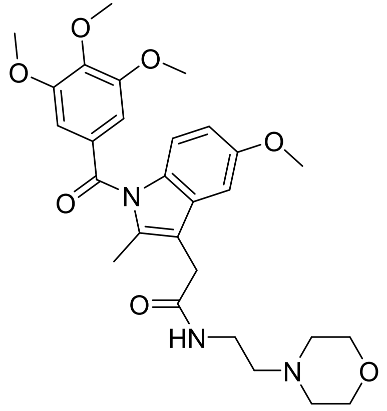	-9.1
**IND-12**	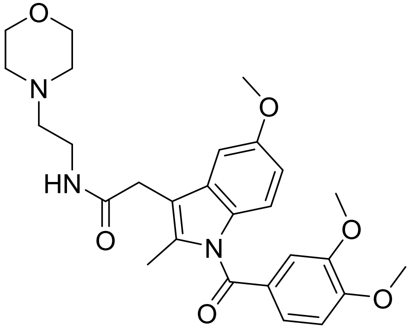	-9.9

**IND-13**	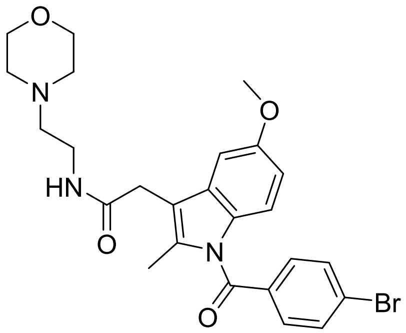	NA
**IND-14**	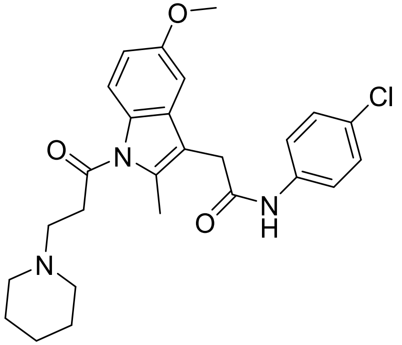	-10.6
**IND-15**	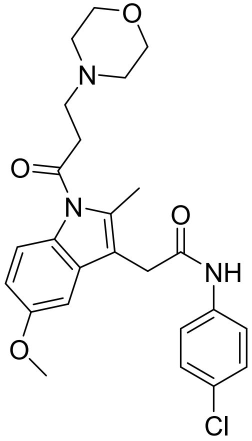	-10.4

**IND-16**	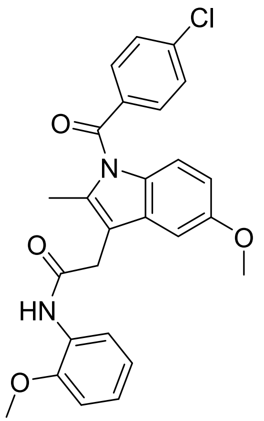	-8.0
**IND-17**	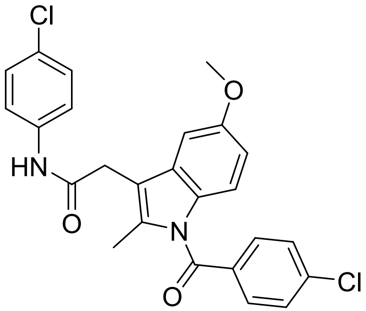	-8.1
**IND-18**	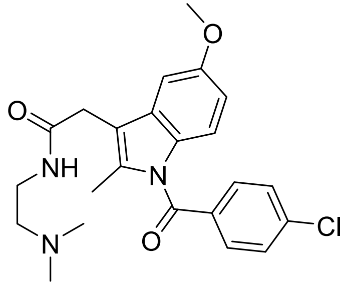	-9.6

**IND-19**	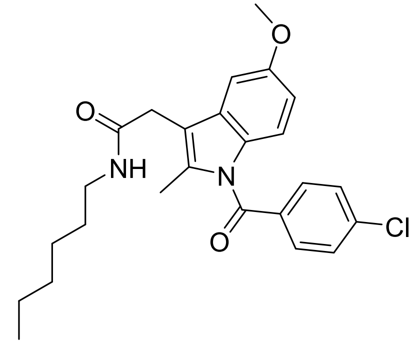	-8.0
**IND-20**	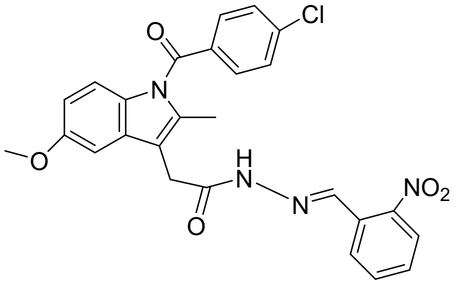	-11.6
**IND-21**	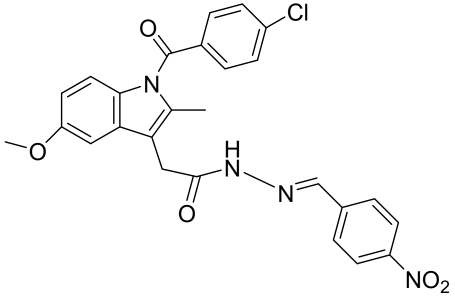	-11.8

**IND-22**	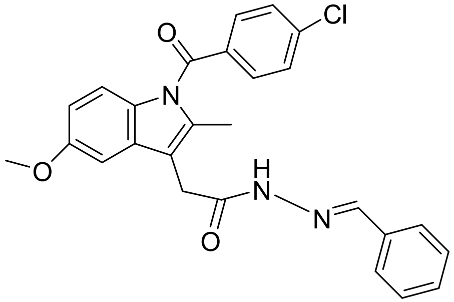	-11.2
**IND-23**	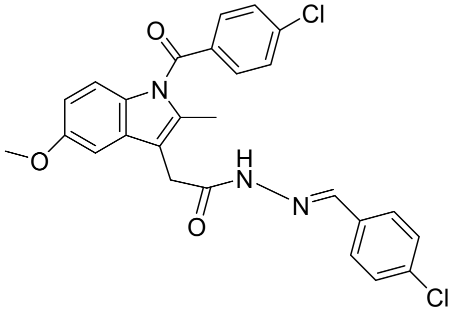	-11.6
**IND-24**	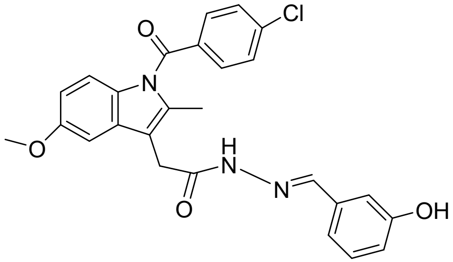	-11.4

**IND-25**	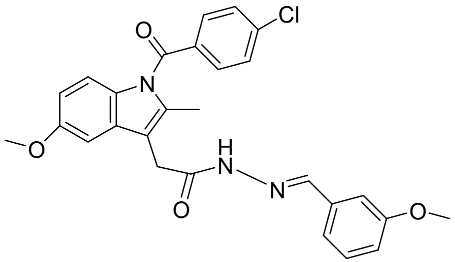	-11.3
**IND-26**	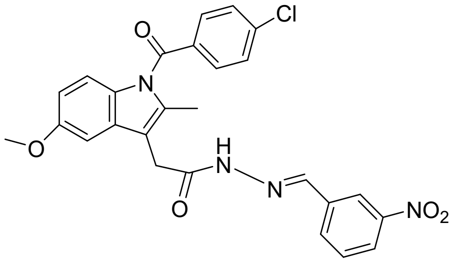	-11.9
**IND-27**	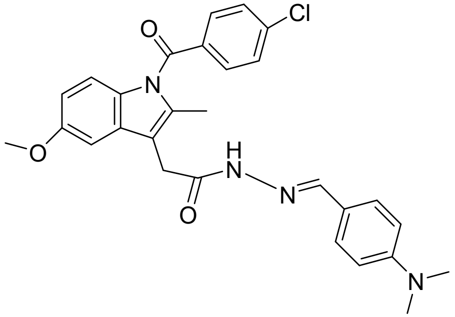	-11.6

**IND-28**	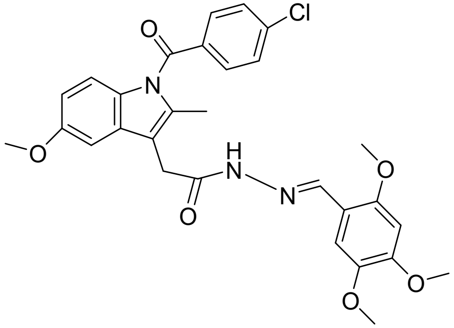	-11.4
**IND-29**	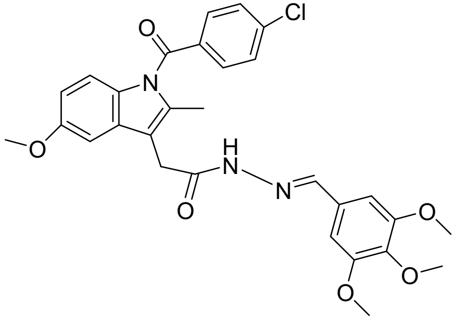	-11.3
**IND-30**	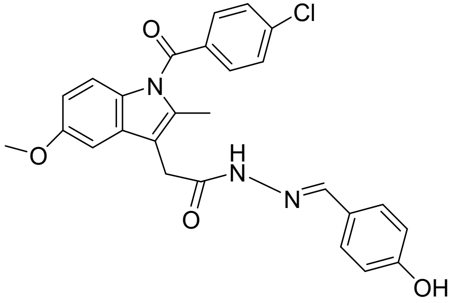	-11.4

**IND-31**	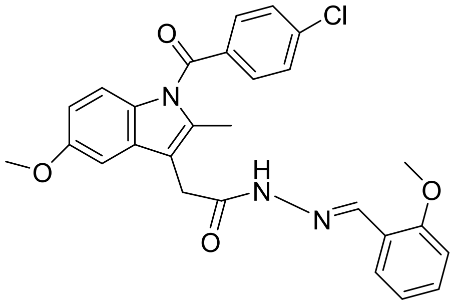	-11.4
**IND-32**	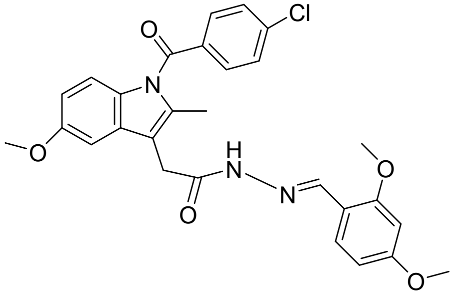	-11.4
**IND-33**	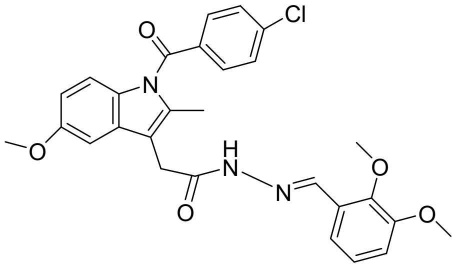	-12.1

**IND-34**	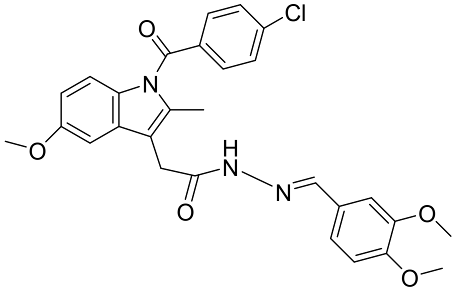	-11.7
**IND-35**	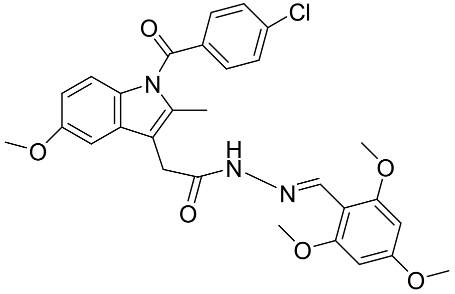	-11.4
**IND-36**	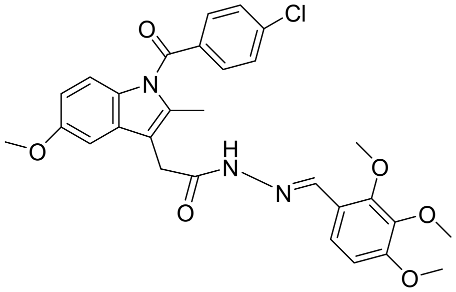	-12.3

**IND-37**	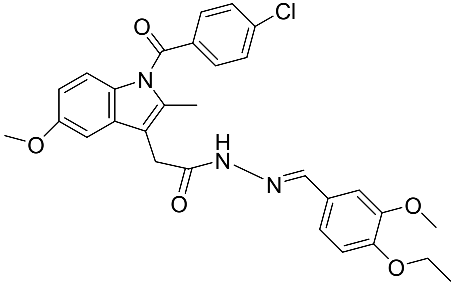	-11.3
**IND-38**	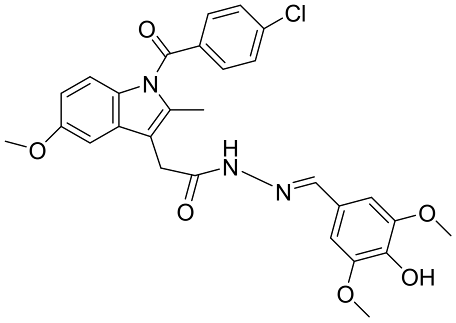	-11.4
**IND-39**	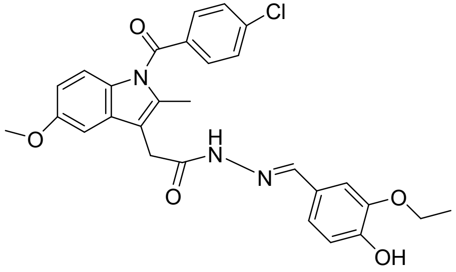	-11.2

**IND-40**	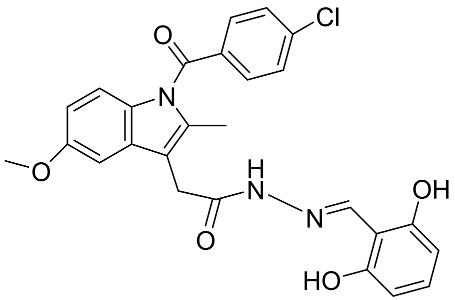	-11.1
**IND-41**	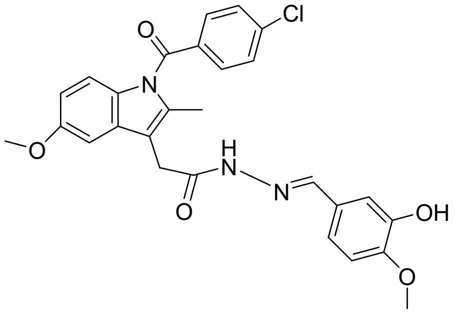	-11.7
**IND-42**	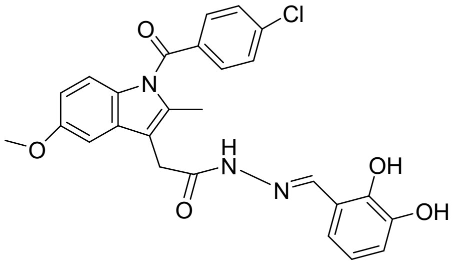	-11.3

**IND-43**	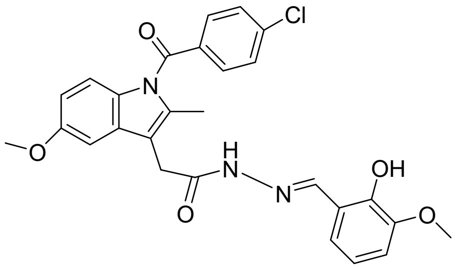	-11.5
**IND-44**	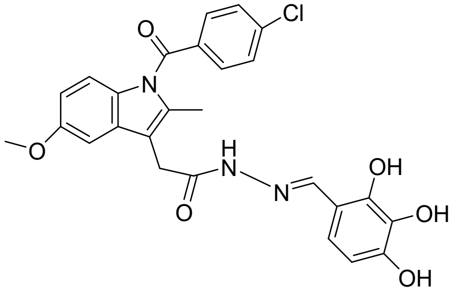	-11.6
**E20 (Bound)**		-11

Table S2. The physiochemical, lipophilicity, water solubility, pharmacokinetics, drug likeliness and toxicity predictions of 44 indomethacin derivatives.

Sr.No	Swiss-ADME												ADMET SAR 2.0				
	Physicochemical properties						lipophilicity	Water Solubility	Pharmakokinetics	Drug Likeliness			Human ether-a-go-go-related gene inhibition	AMES Mutagenesis	Acute oral toxicity (c)	Carcinogenicity (three-class)	Rat Acute Toxicity (LD50)
**Analogue**	**Mwt**	**nrotb**	**HBA**	**HBD**	**MR**	**TPSA (A)**	**Consensus Log Po/w**	**class**	**GI absorption**	**Lipinski**	**Veber**	**Muegge**					
IND	357.8	5	4	1	96.12	68.53	3.63	Moderately Soluble	High	0	0	0	Weak inhibitor	Non-AMES toxic	I	**Danger**	**4.0722**
IND-1	432.90	7	3	1	123.38	60.33	4.8	Poorly soluble	High	1	0	1	Weak inhibitor	Non-AMES toxic	III	**Danger**	2.5705
IND-2	446.93	7	3	1	128.34	60.33	5.13	Poorly soluble	High	1	0	1	Weak inhibitor	Non-AMES toxic	III	**Danger**	2.5368
IND-3	462.92	8	4	1	129.87	69.56	4.78	Poorly soluble	High	0	0	1	Weak inhibitor	Non-AMES toxic	III	**Danger**	2.6481
IND-4	423.5	10	5	1	120.96	72.8	3.12	Moderately Soluble	High	0	0	0	Weak inhibitor	Non-AMES toxic	III	**Non-required**	2.6467
IND-5	453.96	9	4	1	132.43	63.57	4.18	Moderately Soluble	High	0	0	0	Weak inhibitor	Non-AMES toxic	III	**Non-required**	2.4728
IND-6	427.92	9	4	1	121.01	63.57	3.81	Moderately Soluble	High	0	0	0	Weak inhibitor	Non-AMES toxic	III	**Non-required**	2.4756
IND-7	453.53	11	6	1	127.45	82.03	3.05	Moderately Soluble	High	0	1	0	Weak inhibitor	Non-AMES toxic	III	**Non-required**	2.6112

IND-8	483.56	12	7	1	133.94	91.26	3.15	Moderately Soluble	High	0	1	0	Weak inhibitor	Non-AMES toxic	III	**Non-required**	2.6319
IND-9	443.54	9	4	1	131.97	63.57	4.02	Moderately Soluble	High	0	0	0	Weak inhibitor	Non-AMES toxic	III	**Non-required**	2.6762
IND-10	509.59	12	7	1	145.36	91.26	3.49	Moderately Soluble	High	1	1	0	Weak inhibitor	Non-AMES toxic	III	**Non-required**	2.5735
IND-11	525.59	12	8	1	146.44	100.49	2.95	Moderately Soluble	High	1	1	0	Weak inhibitor	Non-AMES toxic	III	**Non-required**	2.5649
IND-12	495.57	11	7	1	139.95	91.26	2.95	Moderately Soluble	High	0	1	0	Weak inhibitor	Non-AMES toxic	III	**Non-required**	2.5009
IND-13	514.41	9	5	1	134.67	72.8	3.54	Moderately Soluble	High	1	0	0	Weak inhibitor	Non-AMES toxic	III	**Non-required**	2.6388
IND-14	467.99	9	4	1	137.24	63.57	4.47	Moderately Soluble	High	0	0	0	Weak inhibitor	Non-AMES toxic	III	**Non-required**	2.5647
IND-15	469.96	9	5	1	133.51	72.8	3.61	Moderately Soluble	High	0	0	0	Weak inhibitor	Non-AMES toxic	III	**Non-required**	2.5521
IND-16	462.92	8	4	1	129.87	69.56	4.8	Poorly soluble	High	0	0	1	Weak inhibitor	Non-AMES toxic	III	**Danger**	2.566
IND-17	467.31	7	3	1	128.39	60.33	5.33	Poorly soluble	High	1	0	1	Weak inhibitor	Non-AMES toxic	III	**Danger**	2.5705
IND-18	427.92	9	4	1	119.48	63.57	3.59	Moderately Soluble	High	0	0	0	Weak inhibitor	Non-AMES toxic	III	**Non-required**	2.7849
IND-19	440.96	11	3	1	126.19	60.33	5.21	Poorly soluble	High	1	1	1	Weak inhibitor	Non-AMES toxic	III	**Non-required**	2.6996
IND-20	504.92	9	6	1	139.15	118.51	4.07	Poorly soluble	Low	1	0	1	Weak inhibitor	AMES toxic	III	Non-required	2.6707
IND-21	504.92	9	6	1	139.15	118.51	4.07	Poorly soluble	Low	1	0	1	Weak inhibitor	AMES toxic	III	Non-required	2.7124
IND-22	459.92	8	4	1	130.33	72.69 Å	4.73	Poorly soluble	High	0	0	1	Weak inhibitor	Non-AMES toxic	III	Non-required	2.7362
IND-23	494.37	8	4	1	135.34	72.69	5.2	Poorly soluble	High	1	0	1	Weak inhibitor	Non-AMES toxic	III	Non-required	2.7362
IND-	475.	8	5	2	132	92.9	4.3	Poorly	High	0	0	1	Weak	Non-AMES	III	Non-	2.91

24	92				.35	2		soluble					inhibitor	toxic		required	62
IND-25	489.95	9	5	1	136.82	81.92	4.68	Poorly soluble	High	0	0	1	Weak inhibitor	Non-AMES toxic	III	Non-required	2.7729
IND-26	504.92	9	6	1	139.15	118.51	4.02	Poorly soluble	Low	1	0	1	Weak inhibitor	AMES toxic	III	Non-required	2.7124
IND-27	502.99	9	4	1	144.54	75.93	4.84	Poorly soluble	High	1	0	1	Weak inhibitor	Non-AMES toxic	III	Non-required	2.6017
IND-28	550	11	7	1	149.81	100.38	4.66	Poorly soluble	High	1	1	1	Weak inhibitor	Non-AMES toxic	III	Non-required	2.7408
IND-29	550	11	7	1	149.81	100.38	4.68	Poorly soluble	High	1	1	1	Weak inhibitor	Non-AMES toxic	III	Non-required	2.7877
IND-30	475.92	8	5	2	132.35	92.92	4.3	Poorly soluble	High	0	0	1	Weak inhibitor	Non-AMES toxic	III	Non-required	2.9162
IND-31	489.95	9	5	1	136.82	81.92	4.75	Poorly soluble	High	0	0	1	Weak inhibitor	Non-AMES toxic	III	Non-required	2.732
IND-32	519.98	10	6	1	143.31	91.15	4.72	Poorly soluble	High	1	0	1	Weak inhibitor	Non-AMES toxic	III	Non-required	2.7366
IND-33	519.98	10	6	1	143.31	91.15	4.77	Poorly soluble	High	1	0	1	Weak inhibitor	Non-AMES toxic	III	Non-required	2.7521
IND-34	519.98	10	6	1	143.31	91.15	4.72	Poorly soluble	High	1	0	1	Weak inhibitor	Non-AMES toxic	III	Non-required	2.8064
IND-35	550	11	7	1	149.81	100.38	4.69	Poorly soluble	High	1	1	1	Weak inhibitor	Non-AMES toxic	III	Non-required	2.7366
IND-36	550	11	7	1	149.81	100.38	4.76	Poorly soluble	High	1	1	1	Weak inhibitor	Non-AMES toxic	III	Non-required	2.7408
IND-37	534	11	6	1	148.12	91.15	5.07	Poorly soluble	High	1	1	1	Weak inhibitor	Non-AMES toxic	III	Non-required	2.8245
IND-38	535.98	10	7	2	145.34	111.38	4.42	Poorly soluble	High	1	0	1	Weak inhibitor	Non-AMES toxic	III	Non-required	2.8571
IND-39	519.98	10	6	2	143.65	102.15	4.74	Poorly soluble	High	1	0	1	Weak inhibitor	Non-AMES toxic	III	Non-required	2.7831
IND-40	491.92	8	6	3	134.38	113.15	4.01	Moderately Soluble	High	0	0	0	Weak inhibitor	Non-AMES toxic	III	Non-required	2.8484

IND-41	505.95	9	6	2	138.85	102.15	4.44	Poorly soluble	High	1	0	1	Weak inhibitor	Non-AMES toxic	III	Non-required	2.8705
IND-42	491.92	8	6	3	134.38	113.15	3.94	Moderately Soluble	High	0	0	0	Weak inhibitor	Non-AMES toxic	III	Non-required	2.8319
IND-43	505.95	9	6	2	138.85	102.15	4.42	Poorly soluble	High	1	0	1	Weak inhibitor	Non-AMES toxic	III	Non-required	2.7957
IND-44	507.92	8	7	4	136.4	133.38	3.53	Moderately Soluble	Low	1	0	1	Weak inhibitor	Non-AMES toxic	III	Non-required	2.8242
